# Socioeconomic and environmental effects of soybean production in metacoupled systems

**DOI:** 10.1038/s41598-021-98256-6

**Published:** 2021-09-20

**Authors:** Ramon Felipe Bicudo da Silva, Andrés Viña, Emilio F. Moran, Yue Dou, Mateus Batistella, Jianguo Liu

**Affiliations:** 1 Center for Systems Integration and Sustainability, Department of Fisheries and Wildlife, Michigan State University, East Lansing, MI 48823 USA; 2 Center for Environmental Studies and Research, State University of Campinas, Campinas, 13083-867 Brazil; 3 Department of Geography, University of North Carolina, Chapel Hill, USA; 4 Center for Global Change and Earth Observations, Michigan State University, East Lansing, MI 48823 USA; 5 Environmental Geography Group, Institute for Environmental Studies, Vrije Universiteit Amsterdam, de Boelelaan, 1081HV Amsterdam, The Netherlands; 6 Embrapa Agricultural Informatics, Brazilian Agricultural Research Corporation, Campinas, 13083-886 Brazil

**Keywords:** Environmental impact, Environmental social sciences, Sustainability

## Abstract

Human–environment interactions within and across borders are now more influential than ever, posing unprecedented sustainability challenges. The framework of metacoupling (interactions within and across adjacent and distant coupled human–environment systems) provides a useful tool to evaluate them at diverse temporal and spatial scales. While most metacoupling studies have so far addressed the impacts of distant interactions (telecouplings), few have addressed the complementary and interdependent effects of the interactions within coupled systems (intracouplings) and between adjacent systems (pericouplings). Using the production and trade of a major commodity (soybean) as a demonstration, this paper empirically evaluates the complex effects on deforestation and economic growth across a globally important soybean producing region (Mato Grosso in Brazil). Although this region is influenced by a strong telecoupling process (i.e., soybean trade with national and international markets), intracouplings pose significant effects on deforestation and economic growth within focal municipalities. Furthermore, it generates pericoupling effects (e.g., deforestation) on adjacent municipalities, which precede economic benefits on adjacent systems, and may occur during and after the soybean production takes place. These results show that while economic benefits of the production of agricultural commodities for global markets tend to be localized, their environmental costs tend to be spatially widespread. As deforestation also occurred in adjacent areas beyond focal areas with economic development, this study has significant implications for sustainability in an increasingly metacoupled world.

## Introduction

Almost all places around the world are currently interconnected through the growing flows of people, goods, capital, and information. While such interconnections have existed for a long time, they have increased drastically in recent decades^1–3^. For example, while over the past three decades the world’s total food production has doubled^4^, a growing fraction of this expansion is associated with commodities produced for global markets^6^. Thus, current dynamics of land-use and land-cover change are being shaped by new forces, such as the ever-increasing demand for globalized commodities^5^, with the result that globalization is now more influential than ever before^1^. This poses unprecedented challenges for the sustainable management of natural resources around the world. The international soybean trade offers an excellent lens through which to explore such challenges.

Soybean has a long history of cultivation for human food and animal feed in China, but since the 1970s, it has been transformed into a commodity crop with industrial uses, particularly in the livestock sector^7^. This sector has grown considerably in response to a dietary shift towards more animal protein due to the increase in economic affluence in China over the last three decades^8^. As a result of this dietary shift, starting in 2002 soybean (and its derivatives) started to be considered an industrial, rather than an agricultural commodity in China, which allowed the liberalization of their importation^9^. The result is that soybean imports, predominantly from the US and Brazil, increased almost exponentially^7^.

**Figure 1:**
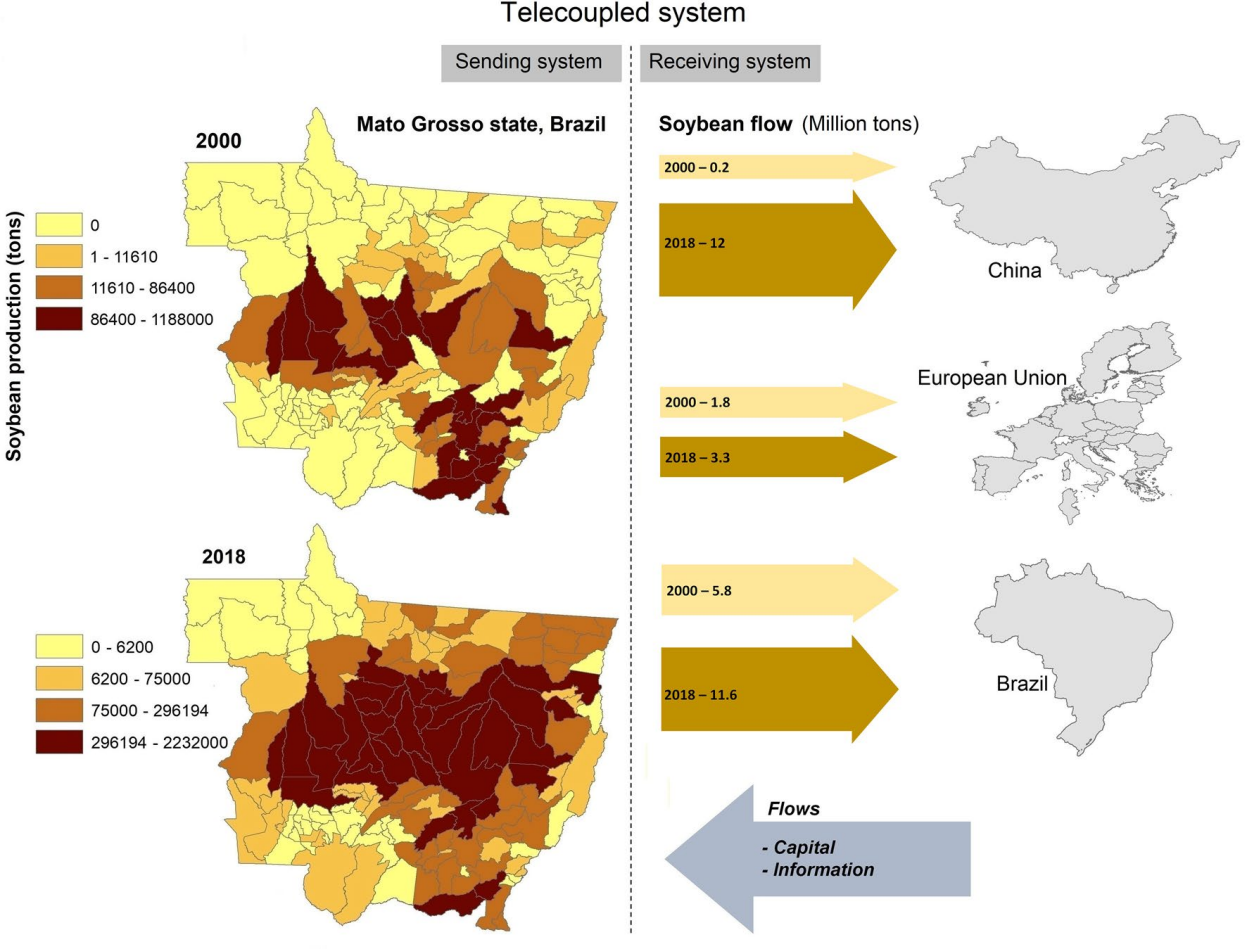
Conceptualization of the telecoupled system, with soybean trade as the major flow. Telecoupling is conceptualized as the flow of soybean between Mato Grosso, Brazil and major international markets (China and the European Union), as well as domestic markets (other states) within Brazil. In 2018 ca. 56% of the state’s total soybean produced went to China and the European Union. In exchange, Mato Grosso received flows of capital and information. Maps were created by the Authors using the software QGIS 3.16.3 (https://qgis.org/en/site/).

The drastic increase in soybean production and export has brought not only socioeconomic effects^10,11^, but also serious environmental consequences, as it has both directly and indirectly induced land-use and land-cover changes in Brazilian biomes^12–15^. Thus, the international trade of soybean constitutes a complex human–environment interaction that can be conceptualized as a metacoupled system^16^. Under this conceptual framework, distant, adjacent, and local interactions may occur simultaneously and complementarily^17,18^. While the metacoupling framework has been used to evaluate the environmental outcomes induced by distant and adjacent interactions such as trade and has been successfully applied to many systems worldwide^18–25^, no empirical studies have been conducted to assess the economic and environmental effects of intracouplings (human–environment interactions such as soybean production within a system) and pericouplings (human–environment interactions among adjacent systems^16^) within the context of a dominant telecoupling process (Fig. 1). To fill this important knowledge gap, this study focused on all the municipalities within the state of Mato Grosso, Brazil (a major producer of soybean for international and national markets^26^), and evaluated socioeconomic and environmental outcomes of the soybean production at the producing municipalities and on neighboring municipalities.

Our rationale reflects the behavior of soybean producers in Brazilian agricultural areas^27,28^, in which land-use decisions are driven by producers’ expectations of economic benefits based on previous years of commodity price and market demand, which in turn stimulates further land conversion^28^. In this regard, our study goes beyond the municipality boundary of the focal production system^27^ to assess how the aggregated pattern of individual producers in one municipality drives intracoupling and pericoupling effects. In regions such as Mato Grosso state in Brazil, soybean production in a given year requires preparation during previous years^27,28^. Furthermore, once soybean production gets started in an area, other dynamics also occur, which may lead to different socioeconomic and environmental outcomes^29^. Therefore, we hypothesize that while soybean production triggers land change processes (e.g., deforestation) not only within but also in neighboring municipalities, associated economic benefits only accrue within the soybean producing municipality. In this study we refer to “intracoupling effects” as those occurring within municipalities while “pericoupling effects” as those occurring in adjacent municipalities.

Previous studies have addressed indirect land use change by taking pixel-based approaches to demonstrate the patterns in which soybean replace pastureland, while leading to the expansion of pastureland in forested areas^30,31^; or how soybean production promotes economic benefits at varying geographical levels^29,32^. However, this study addresses two crucial components of complex metacoupled systems. The first one refers to the interactive relationship between land-use and social process within intracoupled systems, while the second one refers to the pericoupling effects between adjacent systems, both at the municipality level. Here we understand an intracoupled system as a place (e.g., a municipality) that exhibits reciprocal human-nature interactions and feedbacks, while pericoupled systems as adjacent systems connected through flows of people, information, resources, and capital, among others^16^. Such interactions include the use of natural resources to meet human needs (e.g., soybean production) and its socio-environmental effects. Considering the global flows of various natural resources, intracoupled and pericoupled systems may interact with telecoupled systems^1^, which together form metacoupled systems—i.e., an assemblage of coupled systems at different distances and spatial configurations^16^. Understanding the interactions among these coupled systems and their multiple effects is crucial for guiding informed decision-making processes related to socioeconomic development in agricultural frontiers.

## Results

**Figure 2:**
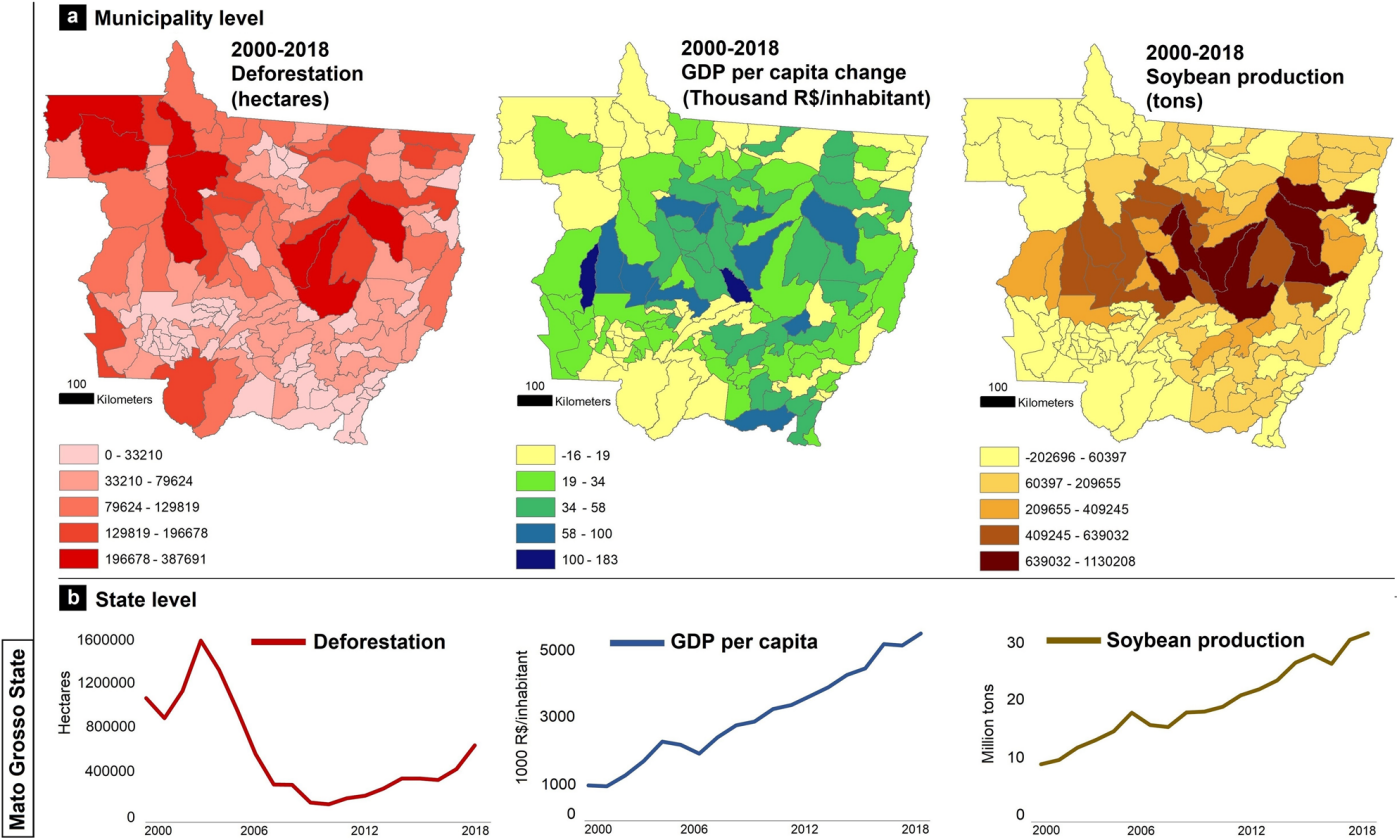
Dynamics of deforestation, Gross Domestic Product (GDP) per capita, and soybean production in Mato Grosso, Brazil, between 2000 and 2018. (**a**) Maps represent the changes observed between 2000 and 2018 in forest cover (i.e., deforestation), GPD per capita, and soybean production in each of the 141 municipalities comprising the state of Mato Grosso, Brazil. (**b**) Line graphs show the trends (2000–2018) of these variables aggregated to the state level. Maps were created by the Authors using the software QGIS 3.16.3 (https://qgis.org/en/site/).

Mato Grosso exhibited a significant agricultural expansion, although it was not spatially uniform throughout the state (Fig. 2a). For instance, while municipalities with the largest soybean production in 2000 also exhibited the largest production in 2018, the municipalities that exhibited a significant increase in their soybean production between 2000 and 2018 tended to be clustered around the largest producers (Fig. 2a and Supplementary Fig. 1). This pattern is crucial for understanding the influence of neighboring municipalities when examining the spatiotemporal effects of intracouplings and pericouplings on environmental and socioeconomic outcomes, such as deforestation and the change in Gross Domestic Product (GDP) per capita, respectively (Fig. 2a,b).

**Figure 3:**
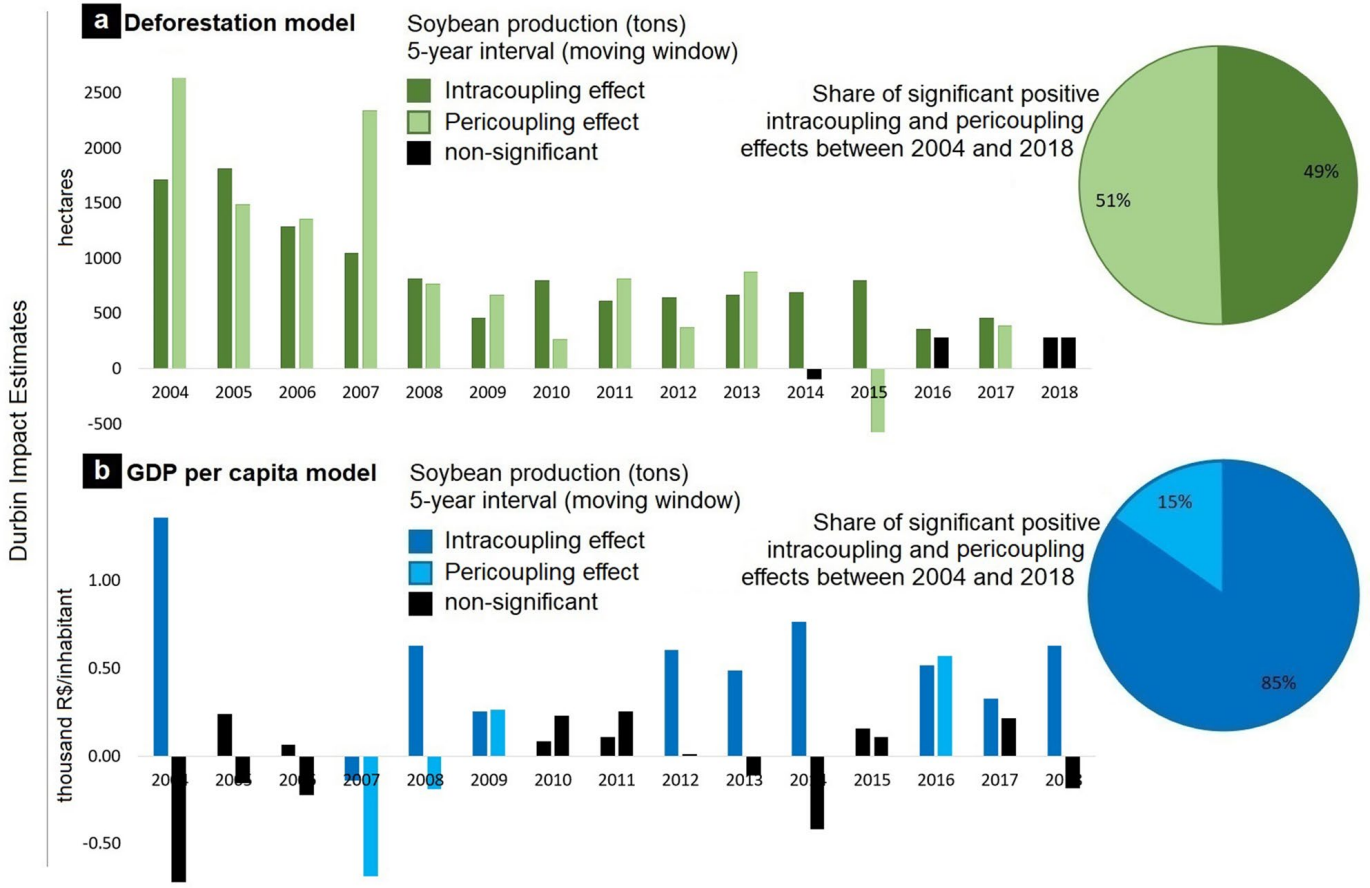
Results of spatial Durbin Error Models (SDEM) for fifteen subsets of model simulations between 2000 and 2018 using a moving window of five years. (**a**) SDEM to account for intracoupling and pericoupling effects of soybean production on deforestation across municipalities in the state of Mato Grosso, Brazil. (**b**) SDEM to account for intracoupling and pericoupling effects of soybean production on Gross Domestic Product (GDP) per capita across municipalities in the state of Mato Grosso, Brazil.

We developed two models to assess the effects of soybean production on environmental (deforestation model) and socioeconomic [Gross Domestic Product (GDP) per capita model] variables within (intracoupling) and in adjacent (pericoupling) municipalities (see "Methods"). Results of the deforestation model show significant intracoupling and pericoupling effects of the changes in soybean production on deforestation (Fig. 3a) at five-year intervals (i.e., moving window, see "Methods"), after controlling for other biophysical and socioeconomic variables [e.g., slope, forest cover, GDP per capita, area under pastureland, and second-crop production (Supplementary information 1)]. These results suggest that throughout the 2000–2018 period, soybean production was significantly and positively correlated with deforestation not only within municipalities (i.e., intracoupling effect) but also across neighboring municipalities (i.e., pericoupling effect), with few exceptions (Fig. 3a and Supplementary information 1). The deforestation model also revealed that the increase in pastureland areas at five-year intervals exhibited a significant positive relationship with deforestation but only within the same municipality and not in adjacent municipalities (Supplementary information 1). Although the increase in pastureland exhibited a significant negative relationship with deforestation in adjacent municipalities during some time periods (e.g., 2010–2014), the intracoupling effect contributed with around 93% of the total significant relationship between the increase in pastureland and deforestation. The observed significant negative pericoupling effect of pasture area increase in few deforestation model subsets (Supplementary information 1) may be explained by the expansion of pastureland in previous years. Hence, the large availability of pastureland during a given period in a set of municipalities discourages the increase in their neighbors, thus negatively affecting the deforestation trend.

After controlling for variables such as slope, deforestation, pastureland, agricultural labor, maize second-crop production, cattle herd, and forest cover (Supplementary information 2), our results of the GDP per capita model show that soybean production was significantly and positively related with the growth of GDP per capita throughout most of the 2000–2018 period, but only within municipalities (i.e., intracoupling effect) (Fig. 3b and Supplementary information 2). These results suggest that soybean production within a municipality was associated with the economic growth of the same municipality, while showing no association (or a weak association) with the economic conditions of neighboring municipalities (i.e., non-pericoupling effect—only 15% of the total positive effects of soybean production associated with an increase on GDP per capita were from soybean production in neighboring municipalities—Fig. 3b). Furthermore, a Mann–Whitney U test revealed that compared to non-soybean producing municipalities, the GDP per capita in municipalities producing soybean was higher throughout the 2000–2018 (Supplementary Fig. 2). Our model results also revealed that during the five-year intervals in which soybean production did not exhibit a significant positive relationship with the growth of GDP per capita within municipalities, the variable ‘maize second-crop production’ did (Supplementary information 2). This suggests that an increase in the production of maize as a second-crop significantly complements the economic growth within a municipality.

Considering the results for Global Moran’s I tests for spatial dependence on the GDP per capita models, which indicated non-spatial dependence (except for years 2005, 2006, and 2007—i.e., p-values < 0.05), ordinary least square (OLS; Supplementary information 3) regression were applied and results confirmed the significance (p-values < 0.05) of soybean production growth as a major variable positively correlated with the local GPD per capita growth at the municipality level. Additionally to our spatial Durbin GDP per capita models based on five-year moving windows, two different sets of seven- and ten-year moving windows were applied to verify if pericoupling effects of GDP per capita growth would be different from zero, but no evidences of such effects were found.

## Discussion

**Figure 4:**
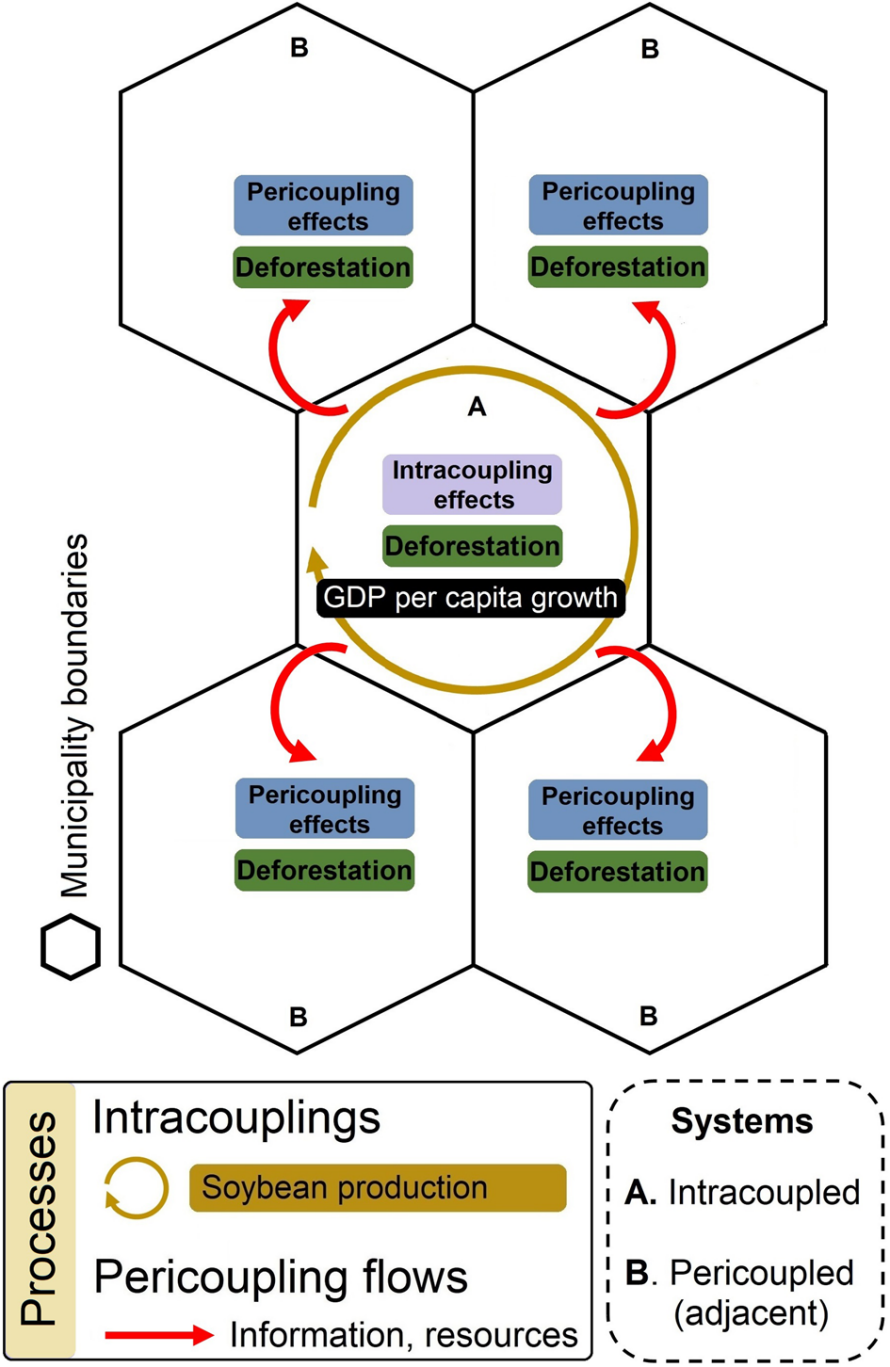
Conceptualization of the intracoupled and pericoupled systems. Intracouplings and pericouplings are conceptualized as the flows of people, information, resources, and capital both within and across neighboring municipalities in response to the soybean market. The pericoupling flows, in turn, have effects on land change dynamics (e.g., pasture conversion, deforestation) in adjacent municipalities.

Our empirical analyses demonstrate that in addition to a strong telecoupling process (i.e., the trade of soybean for national and international markets, Fig. 1) that has been reported to cause significant socioeconomic^11,29,32^ and environmental^12–15^ effects, both intracouplings and pericouplings also exert significant effects (Fig. 4). These interdependent systems include flows of not only soybean but also of information, resources, capital and people, among others^27,28,33^, all of which influence land-use decisions not only locally but also in adjacent municipalities.

In contrast to traditional land change analyses that often treat socioeconomic and environmental outcomes by adopting a specific time interval (e.g., defined within a single period)^34–36^, our analyses evaluate deforestation and economic growth as dynamic processes through the use of a moving window approach, hence yielding fifteen subsets of model simulation. Furthermore, through the use of a Spatial Durbin Error Model (SDEM), these analyses provide a different perspective from previous studies that focused on general indirect land change processes pushing deforestation into far distant areas^37,38^. Hence, our study empirically addressed local (intracoupled) and adjacent (pericoupled) systems simultaneously by taking the municipality as the unit of analysis, and additionally applying the same modeling approach for both processes—economic growth and deforestation—while putting them into the same theoretical metacoupling framework.

The deforestation model shows that the expansion of soybean production was significantly related with deforestation not only within municipalities but also across neighboring municipalities. This is a key empirical finding as it demonstrates that the expansion of soybean production in a given municipality has pericoupling effects that influence land change dynamics in adjacent municipalities within the State of Mato Grosso, hence increasing their likelihood of experiencing deforestation. This land change process is especially reinforced by indirect land-use change in which the expansion of pastureland precedes the expansion of soybean^13,34^, with soybean areas replacing pastureland areas that had driven deforestation in neighboring municipalities. Furthermore, we demonstrated that neighboring municipalities are related regarding land-use decisions, evidencing that agglomeration economies in fact play an important role not only at the local level but for entire frontier regions^39^. As has been previously stated^16^, our analysis demonstrates that human-nature interactions within a system do affect adjacent systems. This result may be particularly explained by the flow of information influencing land-use decisions among neighboring localities—e.g., through the propagation of information in frontier regions^40^. Among major information flows, we highlight the positively correlated local economic growth promoted by soybean expansion (observed by the GDP per capita model), lower land prices in neighboring municipalities with large areas of arable land available^41^, lower integration with international markets and hence not committed with supply chain agreements for sustainability, thus facilitating deforestation^42^, the scarcity of arable lands in former producing municipalities pushing producers to expand into new adjacent areas, and the proximity to more developed hubs of production, which facilitate logistics and trade^43^.

In contrast, the GDP per capita model shows that the expansion of soybean production was significantly related with economic growth but only within municipalities and not across neighboring municipalities (i.e., no significant pericoupling effects associated with economic growth were found). During the period between 2000 and 2018, the production value of the soybean contributed, on average, to around 27% of municipalities’ GDP^44^, which demonstrates a strong contribution to the State’s economy. This result empirically confirms that while large agribusiness development hubs greatly benefit from the production of agricultural commodities, adjacent municipalities do not equally benefit^45^. We highlight that producers in Mato Grosso may purchase farms and open areas for soybean production in municipalities adjacent to well-established production centers, which reinforce economic development in those well-established centers, but not in the neighboring municipalities. This occurs because in these well-established centers producers can more easily: find storage facilities and trading offices; purchase machinery, seeds, and technological infrastructure; and find professional consultants^45^. Thus, municipalities adjacent to large soybean production centers, which constitute pericoupled systems since they are interconnected through many flows, including people (e.g., farmers), information, and capital, among others, experience significantly lower economic benefits than adjacent agribusiness development hubs^45^.

In addition to the broad distribution of environmental costs (e.g., deforestation within and in neighboring municipalities) associated with quite localized economic benefits (e.g., GDP per capita growth within but not in neighboring municipalities) of soybean production found in this study, Mato Grosso has experienced the entrance of large international groups purchasing land for soybean production since 2000^46,47^ (which is negatively perceived by 95% of the producers from APROSOJA—the largest association of soybean producers in Mato Grosso^48^). Thus, Mato Grosso may be experiencing a similar outcome as other agricultural frontier areas such as those in the Chaco and Chiquitano woodlands of Bolivia and Paraguay, respectively. In those areas, new comers from adjacent countries (e.g., Brazil, Argentina) produce agricultural commodities that degrade the local environment without favoring the local economy and instead export the economic gains to investors in distant locations^40^. Hage et al.^47^ also highlight the risks of the economic gains from such production systems being exported to geographically distant systems.

Previous studies have shown that soybean production plays an important role in the economic development of soybean producing regions^31,32,49^, although with varying effects according to the region considered. For instance, soybean production exhibited a positive effect on socioeconomic metrics such as the Gini coefficient for income in the new agricultural frontier of the Amazon biome and MATOPIBA (northeast region within the Cerrado biome^49^), while in Mato Grosso no such effect was found^32^. The authors^32^ attribute these regionalized positive effects to regions such as MATOPIBA because those areas are economically underdeveloped and with a large gap in logistics and infrastructure. This situation has improved over the last couple of decades, hence improving the Gini and other metrics of human wellbeing. Similar to these previous studies, which did not evaluate the effects of soybean production on neighboring localities, our results show that the economic development brought by soybean production is limited to local areas and do not propagate to neighboring municipalities as was observed for deforestation. As noted by Lopes et al.^50^, the soybean development in MATOPIBA has promoted ecological exclusion of customary users (e.g., local people, smallholders) while taking over diverse former land uses, but also excluding those communities (e.g., indigenous, smallholders) from the socioeconomic outcomes of this agricultural activity. However, a Mann–Whitney U test (Supplementary Fig. 2) showed that soybean producing municipalities have significantly (p-values < 0.05) higher GDP per capita compared to non-producing municipalities, suggesting that over the long run neighboring municipalities affected by negative pericoupling effects (such as deforestation) may also benefit economically but over time periods longer than five years. This trend is also reinforced by our OLS results (Supplementary information 3) in which soybean growth was significantly correlated with increases in GDP per capita at the local production level. Hence, from a complex coupled human–environment systems perspective, our results show that the environmental costs of soybean production (measured as land changes) in both local and adjacent systems are driven by economic expectations, which constitute a feedback from previous economic outcomes (economic growth associated with soybean production), while the economic development of adjacent systems is conditioned on actual production (i.e., soybean yields) if those previous land changes are followed by soybean production (Fig. 4).

The metacoupling framework^16^ constitutes a suitable approach for both conceptually and empirically evaluating the influence of proximal and distant interactions on socioeconomic and environmental outcomes. Under the influence of telecoupling processes (Fig. 1), Tobler’s first law of geography, which states that “everything is related to everything else, but near things are more related than distant things”^51^, may be appropriate to explain pericoupling effects on land change processes in adjacent municipalities (Fig. 4) but not on socioeconomic development. Consequently, while socioeconomic development pushed by the agribusiness may be localized, the environmental costs are more broadly distributed. Therefore, the analysis of intracouplings and pericouplings within the context of a strong telecoupling process provides relevant implications for sustainability.

While the soybean agribusiness is in fact a motor of economic and social development^31,32,50^, our study highlights new implications of its associated environmental impacts (e.g., deforestation) and economic benefits (e.g., local GDP per capita growth). If the soybean dynamics influence the amount of newcomers in municipalities neighboring large agribusiness centers (i.e., municipalities with higher levels of agribusiness infrastructure^45^), then it is necessary to ensure the economic development at the newcomers’ local level (i.e., pericoupled systems), instead of favoring economic growth of the large agribusiness centers in detriment to the new emerging production areas. Therefore, if economic development does not spillover across broad regions, Federal and State legislators should design redistributive policies aimed at the development of those fronts of production (i.e., the pericoupled systems), hence returning the potential economic gains to local populations in the emerging production areas. In addition, public policies such as the farm credit should be designed in tandem with other policies (e.g., entrepreneurship policies^52^) to stimulate farmers to purchase production inputs at local levels (instead of purchasing in more developed agribusiness centers) and incentivize the growth of small-to-medium local businesses to meet producers’ demand, while also providing other services to their families while attracting more entrepreneurs.

Our spatial Durbin local econometric models allowed to simultaneously assess the effects of a telecoupling process on local (intracoupled) and adjacent (pericoupled) systems. Nevertheless, this methodological approach has limitations with respect to the geographical scope (i.e., looking at Queen 1st-order of contiguity adjacent systems) and thus require complementary studies using different geographic distances^37^, spatial structures^53–55^, and modelling approaches. In addition, as the pericoupling effects on economic growth may only manifest when soybean production follows land changes, they may be influenced by significant and long temporal-lag effects. Therefore, future studies exploring economic development in pericoupled systems, not only in our study area but also in many regions around the world, would benefit by time series analyses over longer temporal periods than those analyzed in this study. Such analyses would better inform socioeconomic policies in agricultural development areas. Furthermore, while our study considered the role of agribusiness centers as drivers of economic development, we mainly focused on how the expansion of soybean production in a given municipality influenced not only itself but also its neighbors. Therefore, future studies should evaluate how metacoupled systems are influenced by large socioeconomic players, such as large agribusiness conglomerates.

## Methods

To assess the effects of intracouplings and pericouplings in a large soybean metacoupled system^16^ on socioeconomic and environmental outcomes, we used the Spatial Durbin Error Model (SDEM) based standard proposed by Vega and Elhorst^56^ that model selection should be done under the context of empirical applications. This model was chosen because it accounts for causal variable attributions at the same cross-section (hereafter intracoupling effects) in 141 municipalities (which are used as units of analysis), while also accounting for causal variable attributions on neighboring municipalities (hereafter “pericoupling effects”)^57,58^. The model provides quantitative estimates of the magnitude of both intracoupling and pericoupling effects and their statistical significance when they are not equal to zero^58^.

Our deforestation and GDP per capita models were designed to answer whether soybean production drives deforestation and changes in GDP per capita in a constant way or if their significance changes over time. Furthermore, they allow assessing whether the effects of soybean production are contained within the producer municipality or if they influence its neighbors in a contagious path dependence pattern^43^.

The soybean production of a given year requires previous years of preparation, which involves soil preparation, land-use and land-cover (LULC) changes, access to credit and financial resources, labor, thereby a set of situations to favor its success^27,28,33^. However, after the crop starts developing in a given area and year, other dynamics are expected to occur as a consequence, which may lead to different socioeconomic and environmental outcomes^31^. Given Tobler’s first law of geography^51^ we hypothesized that the effects of the soybean production in a region will influence neighboring areas through path dependencies. Hence, the intracoupling effects of economic development based on soybean production leads to land changes (e.g., deforestation) in neighbor municipalities (Fig. 4) in the following years, as new producers will expect future gains from their soybean production. Therefore, we should expect pericoupling effects for land changes but not for economic development, which is conditioned by the actual production at the focal system. We chose municipalities as our unit of analysis, given that this constitutes the lowest unit of analysis currently available. While previous studies have shown indirect effects of soybean production on deforestation in frontier areas^37,38^ they did not evaluate cross-sectional units of analysis, hence did not look at intracoupling and pericoupling effects simultaneously and on both land change and economic development processes. The publicly available dataset applied in this research is presented in Supplementary Table 1.

### Moving window approach

Based on a temporal moving window of five years, our models assess the dynamic effects of contemporary changes in soybean production, land use and socioeconomic dimensions on deforestation and GDP per capita. For this purpose, the *yΔ*_*t5*_ represents the dependent variable value of five-year periods (Δ*t5* = *5 years deforestation* or *GDP per capita change*), while the explanatory variables *x*_*t0*_, *x*_*t1*_, *x*_*t5*_, where *t0* representing time invariant variables, *t1* the variable value at the beginning of the period, and *t5* accounting for the change in value over the five-year period (Δ*t5*). This approach has the following advantages of conventional methods: (1) allows assessing the temporal variability, (2) incorporate stochastic process, and (3) and deals with cointegration. Hence, our model approach explores temporal variability within a time period of analysis (e.g., 2000–2018) instead of looking at the entire period of analysis^59^ avoiding issues of convolution and non-stationarity^60^. The approach also allows one to incorporate stochastic processes into non-stationary time series, permitting the study of contemporary effects—i.e., short-term dynamics. Furthermore, by choosing moving windows of 5-year interval (15 in total for each model), this approach allowed one to smooth short-term fluctuations while also allowing the observation of long-term trends. Although many models consider stationarity in time series, stochastic (non-stationary) processes may occur and sometimes yield spurious regressions^61,62^. Since over the period of evaluation (2000–2018) the data time series of the present study may be non-stationary and lack cointegration among variables, our moving window approach is justified, since it allows cointegration between non-stationary variables, thus obtaining non-biased linear regression models^63^.

### Dependent variable of deforestation model

Since 2017, data on the Brazilian LULC is systematically produced by MapBiomas (https://mapbiomas.org/), a multi-institutional initiative aiming at developing annual national LULC data at 30-m spatial resolution from 1985 to the present, through the automatic classification of satellite imagery. The present study uses the collection *v.4.1,* which has a global accuracy of 90%^64^. The original data encompass twenty-seven LULC classes (Level 2) and for the purpose of this study, the class ‘natural forest cover’, key to calculating deforestation (i.e., dependent variable), is represented by the sum of ‘Forest formation’ and ‘Savanna formation’ from MapBiomas. Deforestation was then calculated by the total deforestation in a moving window (*Δt5*) at the municipality level. When deforestation is not observed in a given municipality and period (sometimes showing growth of natural forest cover), we set the value to zero, hence modeling just deforestation dynamics.

### Dependent variable of GDP per capita model

The Gross Domestic Product (in Brazilian Real) is a measure of the total monetary value added by the sectors of agriculture, industry, services and public administration at the municipality level and calculated on an annual basis^65^. The dependent variable ‘GPD per capita change’ (i.e., *Δt5*) is calculated by the municipality’s GPD divided by the respective number of inhabitants in the same year (*GDPpercapita* = *GPD/inhabitants*). Population statistics were obtained from the IBGE Population Estimates service^66^. In addition to the five-year moving windows, for the GDP per capita we also built models with windows of 7 and 10 years to assess for temporal-lag effects.

### Explanatory and control variables: deforestation model

Soybean production was considered a key explanatory variable of interest in our models, as it represents a major agribusiness activity in the state of Mato Grosso—i.e., key to understanding agricultural expansion and land changes. Hence, this variable is represented by ‘soybean production’ measured in tons. The initial ‘natural forest cover’—*t1* (hectares) of each deforestation period is also included. The class ‘pasture’ (in hectares) used in the model corresponds to the ‘pasture’ class from the MapBiomas dataset. While agricultural intensification, a process expected to spare land from agricultural expansion and hence decrease deforestation trends^67–69^ has being observed by previous studies^69,70^, rebound effects may imply that higher yields from agricultural intensification instead of sparing land for conservation would foster more crop expansion (through deforestation and pasture conversion) given the great potential for economic returns^71^. Hence, our model accounts for ‘maize second-crop production’ (tons). Although indirectly, this approach allows evaluating the impacts of agricultural intensification^70^. Our model also considered GPD per capita since economic development and growth may foster land conversion and environmental degradation^72^. Topography was also considered an important factor as it drives large-scale agricultural production^73^. In particular, the variable ‘slope’ represents the mean value (percentage of slope) for each municipality. Slope information was derived from the Shuttle Radar Topography Mission (SRTM) elevation maps developed by Weber et al.^74^. All variables used in each model subset represent the change at time *Δt5* and the value at *t1*, except ‘slope’, which is time invariant (*t0*) and maize second-crop, which is only represented by *Δt5* because as long as this crop is dependent on soybean production, their initial time will always be spatially correlated.

### Explanatory and control variables: GDP per capita model

In addition to the variables described in the deforestation model, the GDP per capita model also incorporated two additional variables: Formal ‘agricultural labor’ (number of laborers employed in the agribusiness) and ‘cattle herd’ (number of animals). The agricultural labor was obtained from the Annual List of Social Information (RAIS in Portuguese), a database of the Ministry of Labor and Employment (MTE in Portuguese)^75^. The cattle herd was obtained from the IBGE Municipal Cattle Survey, which is a systematic annual census of properties in the Brazilian countryside^76^.

### Spatial durbin error model

To evaluate pericoupling effects of municipalities on its neighbors, we used the Spatial Durbin Error Model (SDEM), which accounts for the spatial lag of independent variables^77^. The SDEM subsumes the Spatial Error model (SEM, Eq. 1) and the Spatial Lag of X model (SLX, Eq. 2), and described by (Eq. 3)^57^:

1 $$ y  =  X\beta +  u $$

2 $$ y = X\beta {1 } + W \, X\beta {2 } + \varepsilon $$

3 $$ y = X\beta {1 } + W \, X\beta {2 } + u $$

$$\begin{aligned}&u = \lambda Wu + \varepsilon \\ &\varepsilon \sim N(0, {\sigma }_{\varepsilon }^{2} {I}_{N})\end{aligned}$$

where *β*2 is the cross-partial derivative or indirect effects (i.e., pericoupling) of the neighboring regions in the *W* matrix, and *β*1 the own-region direct effect (i.e., intracouplings). When results from cross-partial derivatives are not equal to zero, it implies impact on neighboring municipalities without endogenous feedback effects, which indicates true local spatial lag^58^. Hence, we deal with cross-sectional dependence with a balanced data of 141 cross-sectional units (*i* = municipalities). *ɛ* is the disturbance vector of random errors from the regression model, which considers an autoregressive spatial process in the error term for the spatial model, where *λ* the autoregressive coefficient of the error terms; while *u* is a vector of independent and identically distributed error terms^78–80^. As ordinary least-squares regression (OLS) is unsuitable for spatial regression models because it assumes independence among observations^60,81^, the proposed SDEM relies on the maximum likelihood estimation^77^, suitable to estimate the significance and magnitude of spatial lags^59,81^. Therefore, spatial lags in the proposed model approach constitute the pericoupling effects of independent variables *X* of a given municipality *j* over the dependent variable *y* in the neighboring municipality *i*. This rationale resembles the flows and mutual feedbacks between municipalities as contextualized in Fig. 4. The spatial weights matrix (*W*) at the municipality level was created by defining a neighbor based on the Queen 1st-order of contiguity approach, adjacency matrix^82,83^. As the SDEM does not yield traditional correlation estimates such OLS regression, the independent explanatory variables’ effects are measured by the average impacts (direct, indirect, and total) and their statistical significances estimated by the Bayesian Markov Chain Monte Carlo method, using the random walk Metropolis algorithm set with 500 simulations^83^. For further model interpretation, see LeSage^77^.

### Statistical analysis

To all subsets of the deforestation and GDP per capita models (fifteen subsets each), all the explanatory variables were tested by the variation inflation factor (VIF). The study considered a variable critical for multicollinearity if VIF > 5.0^84^. To each moving window (model subset) we applied an OLS model and tested its residuals with the Global Moran’s I^85^ to check overall spatial autocorrelation where the null-hypothesis indicates no spatial autocorrelation. A cluster and outlier analysis based on the Anselin Local Moran’s I^86^ was then applied to evaluate clustering patterns over the spatial distribution among municipalities for the overall changes of the three major variables of interest (i.e., GDP per capita, deforestation, and soybean production). To test the assumption of non-stationarity in the time series, we applied the augmented Dickey-Fuller (ADF) test over our dependent and independent variables (performed with aggregated data at State level)^87^. The null-hypothesis indicates non-stationarity, which means the variable data has unit root and the time series data follows a certain trend. To complement the analysis, the Engle-Granger cointegration test was applied by using the ADF assessed cointegration between dependent and independent variables^88^. To check model restrictions the Likelihood Ratio test^89^ was also applied to each of the models’ subsets, to verify if the SDEM has a better fit than an OLS. This test assumes that restricted coefficients are like zero (null-hypothesis), indicating that a less complex model is more suitable, thus suggesting that restrictions are true and a simpler model (e.g., OLS) may have a better fit. Finally, the non-parametric Mann–Whitney U test was applied (normal distribution of data was rejected by a Shapiro–Wilk test using statistical significance threshold of p-value < 0.01^90^) to the GDP per capita in 2000, 2005, 2010, 2015, and 2018, to verify significant statistical differences in GDP per capita values between soybean producing and non-producing municipalities. Statistical tests’ results for model specifications are presented in Supplementary Information 4.

## Supplementary Information

Supplementary Information.
