# Supplementary material for: Socioeconomic and environmental effects of soybean production in metacoupled systems
Source: Sci Rep. 2021 Sep 20;11:18662. doi: 10.1038/s41598-021-98256-6 (PMC8452730; doi:10.1038/s41598-021-98256-6)
Supplement: Supplementary file 1 — Supplementary Information. [file 41598_2021_98256_MOESM1_ESM.pdf]

SUPPLEMENTARY INFORMATION

Complex effects of food commodity production across a metacoupled human-environment system

Ramon Felipe Bicudo da Silva[1, 2], Andrés Viña[1,3], Emilio F. Moran[4], Yue Dou[5], Mateus Batistella[2, 6], Jianguo Liu[1]

[1]Center for Systems Integration and Sustainability, Department of Fisheries and Wildlife, Michigan State University, East Lansing, MI 48823, United States of America, r114857@dac.unicamp.br, liuji@msu.edu

[2]Center for Environmental Studies and Research, State University of Campinas, Campinas 13083-867, Brazil

[3]Department of Geography, University of North Carolina, Chapel Hill, United States of America, vina@msu.edu

[4]Center for Global Change and Earth Observations, Michigan State University, East Lansing, MI 48823, United States of America, moranef@msu.edu

[5]Environmental Geography Group, Institute for Environmental Studies, Vrije Universiteit Amsterdam, de Boelelaan, 1081HV Amsterdam, The Netherlands, yue.dou@vu.nl

[6]Embrapa Agricultural Informatics, Brazilian Agricultural Research Corporation, Campinas 13083-886, Brazil, mateus.batistella@embrapa.br

SUPPLEMENTARY INFORMATION 1

Deforestation models. Results from the Spatial Durbin Error Model (SDEM)

For a better understand of each model's subset result, we firstly present a list with the full names and abbreviations of each variable as follow:

Slope = variable representing topography and is a time invariant information in the model

FCT1 = natural forest cover at the first year of the model subset simulation (e.g., FC of 2000, model subset of 2000—2004)

PastT1 = pastureland area at the first year of the model subset simulation

PastT5 = change in pastureland area over the five-year period ($\Delta t5$)

SoyPT1 = soybean production at the first year of the model subset simulation

SoyPT5 = change in soybean production over the five-year period ($\Delta t5$)

GDPcpT1 = GDP per capita at the first year of the model subset simulation

GDPcpT5 = change in GDP per capita over the five-year period ($\Delta t5$)

SecMT5 = maize second-crop production change over the five-year period ($\Delta t5$)

All variables with "lag." represent the indirect (spillover) effect of the respective variable on its neighbors.

Deforestation model of 2004 (period of 2000—2004)

Coefficients: (asymptotic standard errors)

Variables	Estimate	Std. Error	z value	Pr(>|z|)
(Intercept)	1129700.0000	55047000.0000	0.0205	0.9836
Slope	-1746600.0000	6046300.0000	-0.2889	0.7727
FCT1	133.1200	41.4100	32146.0000	0.0013
PastT1	-40.3610	153.6800	-0.2626	0.7928
PastT5	8452.8000	777.4800	108720.0000	< 2.2e-16
soyPT1	9.2962	107.3300	0.0866	0.9310
SoyPT5	1711.3000	235.4000	72700.0000	0.0000
GDPcpT1	1639100.0000	1842100.0000	0.8898	0.3736
GDPcpT5	-2446300.0000	972730.0000	-25148.0000	0.0119
SecMT5	2411.4000	528.0000	45670.0000	0.0049
lag.Slope	7431600.0000	7631200.0000	0.9738	0.3301
lag.FCT1	-85.2810	72.1300	-11823.0000	0.2371
lag.PastT1	241.0800	270.2700	0.8920	0.3724
lag.PastT5	269.4800	1269.0000	0.2124	0.8318
lag.soyPT1	-499.5100	351.6700	-14204.0000	0.1555
lag.SoyPT5	2631.5000	494.1900	53249.0000	0.0001
lag.GDPcpT1	-9368500.0000	5114700.0000	-18317.0000	0.0670
lag.GDPcpT5	-3574200.0000	2479700.0000	-14413.0000	0.1495
lag.SecMT5	6198.3000	1702.2000	36413.0000	0.0003

Significance of the impact of variables: **p-values**

Variables	Direct	Indirect	Total
Slope	0.7727	0.3301	0.2227
FCT1	0.0013	0.2371	0.4859
PastT1	0.7928	0.3724	0.4235
PastT5	< 2.22e-16	0.8318	0.0000
soyPT1	0.9310	0.1555	0.1769
SoyPT5	0.0000	0.0010	0.0000
GDPcpT1	0.3736	0.0670	0.1608
GDPcpT5	0.0119	0.1495	0.0301
SecMT5	0.0495	0.0003	0.0197

Deforestation model of 2005 (period of 2001—2005)

Coefficients: (asymptotic standard errors)

Variables	Estimate	Std. Error	z value	Pr(>|z|)
(Intercept)	-40493000.0000	65405000.0000	-0.6191	0.5358
Slope	-2886900.0000	5721600.0000	-0.5046	0.6139
FCT1	172.0900	43.7260	39355.0000	0.0830
PastT1	-111.9200	142.5600	-0.7851	0.4324
PastT5	8147.6000	763.3800	106731.0000	< 2.2e-16
soyPT1	-275.5700	112.2500	-24550.0000	0.0141
SoyPT5	1815.1000	195.3400	92921.0000	< 2.2e-16
GDPcpT1	2339100.0000	3008700.0000	0.7774	0.4369
GDPcpT5	-2046100.0000	1830600.0000	-11177.0000	0.2637
SecMT5	-234.7700	670.9500	-0.3499	0.7264
lag.Slope	7987500.0000	7794700.0000	10247.0000	0.3055
lag.FCT1	-50.4080	78.6330	-0.6411	0.5215
lag.PastT1	263.3400	267.2100	0.9855	0.3244
lag.PastT5	266.6900	1278.5000	0.2086	0.8348
lag.soyPT1	-414.6800	318.9700	-13001.0000	0.1936
lag.SoyPT5	1490.2000	420.8200	35411.0000	0.0004
lag.GDPcpT1	-7775100.0000	8101100.0000	-0.9598	0.3372
lag.GDPcpT5	1103600.0000	3219900.0000	0.3427	0.7318
lag.SecMT5	-275.6300	1613.8000	-0.1708	0.8644

Significance of the impact of variables: **p-values**

Variables	Direct	Indirect	Total
Slope	0.6139	0.3055	0.3329
FCT1	0.8303	0.5215	0.1008
PastT1	0.4324	0.3244	0.5669
PastT5	< 2.22e-16	0.8348	0.0000
soyPT1	0.0141	0.1936	0.0476
SoyPT5	< 2.22e-16	0.0004	0.0000
GDPcpT1	0.4369	0.3372	0.5348
GDPcpT5	0.2637	0.7318	0.7543
SecMT5	0.7264	0.8644	0.7583

Deforestation model of 2006 (period of 2002—2006)

Coefficients: (asymptotic standard errors)

Variables	Estimate	Std. Error	z value	Pr(>|z|)
(Intercept)	-19335000.0000	53655000.0000	-0.3604	0.7186
Slope	-4094500.0000	5639500.0000	-0.7260	0.4678
FCT1	175.5600	43.7100	40165.0000	0.0591
PastT1	-17.6060	132.3900	-0.1330	0.8942
PastT5	8080.5000	747.7100	108069.0000	< 2.2e-16
soyPT1	103.1200	99.3850	10376.0000	0.2995
SoyPT5	1289.8000	205.8100	62670.0000	0.0000
GDPcpT1	2297100.0000	1623600.0000	14149.0000	0.1571
GDPcpT5	-2064700.0000	2093800.0000	-0.9861	0.3241
SecMT5	-312.8600	539.5300	-0.5799	0.5620
lag.Slope	6489800.0000	7213700.0000	0.8997	0.3683
lag.FCT1	-81.4340	74.5020	-10931.0000	0.2744
lag.PastT1	122.8600	244.0600	0.5034	0.6147
lag.PastT5	862.4900	1196.0000	0.7211	0.4708
lag.soyPT1	62.1030	190.0400	0.3268	0.7438
lag.SoyPT5	1359.2000	385.6100	35250.0000	0.0004
lag.GDPcpT1	-3087300.0000	4842500.0000	-0.6375	0.5238
lag.GDPcpT5	3867500.0000	3734300.0000	10357.0000	0.3004
lag.SecMT5	750.5500	1368.4000	0.5485	0.5834

Significance of the impact of variables: **p-values**

Variables	Direct	Indirect	Total
Slope	0.4678	0.3683	0.5992
FCT1	0.5906	0.2744	0.1531
PastT1	0.8942	0.6147	0.6420
PastT5	< 2.22e-16	0.4708	< 2.22e-16
soyPT1	0.2995	0.7438	0.4015
SoyPT5	0.0000	0.0004	0.0000
GDPcpT1	0.1571	0.5238	0.8782
GDPcpT5	0.3241	0.3004	0.5891
SecMT5	0.5620	0.5834	0.7695

Deforestation model of 2007 (period of 2003—2007)

Coefficients: (asymptotic standard errors)

Variables	Estimate	Std. Error	z value	Pr(>|z|)
(Intercept)	29574000.0000	41406000.0000	0.7142	0.4751
Slope	-1897700.0000	4596200.0000	-0.4129	0.6797
FCT1	181.9800	34.6730	52484.0000	0.0002
PastT1	49.6230	103.9500	0.4774	0.6331
PastT5	5814.1000	589.7100	98592.0000	< 2.2e-16
soyPT1	164.8800	81.6170	20202.0000	0.0434
SoyPT5	1049.6000	215.6200	48677.0000	0.0011
GDPcpT1	-723650.0000	1020900.0000	-0.7088	0.4784
GDPcpT5	-656940.0000	2059000.0000	-0.3191	0.7497
SecMT5	-833.7700	463.7900	-17977.0000	0.0722
lag.Slope	1240500.0000	5756400.0000	0.2155	0.8294
lag.FCT1	-77.7310	58.8980	-13198.0000	0.1869
lag.PastT1	-132.4400	189.1800	-0.7001	0.4839
lag.PastT5	2089.2000	1056.9000	19768.0000	0.0481
lag.soyPT1	737.9000	172.9100	42675.0000	0.0198
lag.SoyPT5	2340.8000	448.0600	52243.0000	0.0002
lag.GDPcpT1	-3684000.0000	2272800.0000	-16209.0000	0.1050
lag.GDPcpT5	16422000.0000	4053000.0000	40517.0000	0.0508
lag.SecMT5	-3222.0000	1140.4000	-28252.0000	0.0047

Significance of the impact of variables: **p-values**

Variables	Direct	Indirect	Total
Slope	0.6797	0.8294	0.8454
FCT1	0.0015	0.1869	0.0560
PastT1	0.6331	0.4839	0.6343
PastT5	< 2.22e-16	0.0481	< 2.22e-16
soyPT1	0.0434	0.1977	0.0008
SoyPT5	0.0113	0.0017	0.0000
GDPcpT1	0.4784	0.1050	0.0399
GDPcpT5	0.7497	0.5084	0.4344
SecMT5	0.0722	0.0047	0.0013

Deforestation model of 2008 (period of 2004—2008)

Coefficients: (asymptotic standard errors)

Variables	Estimate	Std. Error	z value	Pr(>|z|)
(Intercept)	18158000.0000	31047000.0000	0.5849	0.5586
Slope	-1513400.0000	2963800.0000	-0.5106	0.6096
FCT1	104.1300	21.6920	48001.0000	0.0016
PastT1	8.2233	66.1720	0.1243	0.9011
PastT5	6304.5000	411.2500	153300.0000	< 2.2e-16
soyPT1	-43.7730	63.5900	-0.6884	0.4912
SoyPT5	818.3700	133.2800	61404.0000	0.0000
GDPcpT1	53210.0000	444520.0000	0.1197	0.9047
GDPcpT5	-1127000.0000	1186500.0000	-0.9498	0.3422
SecMT5	-19.7850	181.2900	-0.1091	0.9131
lag.Slope	1063800.0000	3755600.0000	0.2833	0.7770
lag.FCT1	-8.1499	37.4710	-0.2175	0.8278
lag.PastT1	-4.9570	122.7300	-0.0404	0.9678
lag.PastT5	505.8600	792.6600	0.6382	0.5234
lag.soyPT1	-64.3390	144.4100	-0.4455	0.6559
lag.SoyPT5	769.8400	292.0100	26363.0000	0.0084
lag.GDPcpT1	-771640.0000	1149300.0000	-0.6714	0.5020
lag.GDPcpT5	6302200.0000	3286600.0000	19176.0000	0.0552
lag.SecMT5	-214.7000	480.0900	-0.4472	0.6547

Significance of the impact of variables: **p-values**

Variables	Direct	Indirect	Total
Slope	0.6096	0.7770	0.8584
FCT1	0.0159	0.8278	0.0063
PastT1	0.9011	0.9678	0.9780
PastT5	< 2.22e-16	0.5234	< 2.22e-16
soyPT1	0.4912	0.6559	0.5396
SoyPT5	0.0000	0.0084	0.0195
GDPcpT1	0.9047	0.5020	0.5518
GDPcpT5	0.3422	0.0552	0.1697
SecMT5	0.9131	0.6547	0.6774

Deforestation model of 2009 (period of 2005—2009)

Coefficients: (asymptotic standard errors)

Variables	Estimate	Std. Error	z value	Pr(>|z|)
(Intercept)	4157400.0000	33379000.0000	0.1246	0.90088
Slope	745440.0000	2343400.0000	0.3181	0.75041
FCT1	66.8720	16.8500	39687.0000	7.23E-02
PastT1	11.9240	53.2840	0.2238	0.82292
PastT5	5245.4000	398.5200	131621.0000	< 2.2e-16
soyPT1	-41.3770	31.5670	-13108.0000	0.18994
SoyPT5	457.6400	141.9000	32250.0000	0.00126
GDPcpT1	535270.0000	415070.0000	12896.0000	0.19719
GDPcpT5	2154800.0000	978640.0000	22018.0000	0.02768
SecMT5	73.1510	55.0560	13287.0000	0.18395
lag.Slope	-4534600.0000	3146300.0000	-14412.0000	0.14952
lag.FCT1	8.9457	30.1810	0.2964	0.76692
lag.PastT1	-31.0070	105.5700	-0.2937	0.76898
lag.PastT5	-53.8500	843.3800	-0.0638	0.94909
lag.soyPT1	-139.0400	67.9180	-20472.0000	0.04063
lag.SoyPT5	672.4400	318.4300	21117.0000	0.03471
lag.GDPcpT1	1843000.0000	1095800.0000	16819.0000	0.09259
lag.GDPcpT5	6520800.0000	2644300.0000	24660.0000	0.01366
lag.SecMT5	58.5000	150.9500	0.3876	0.69835

Significance of the impact of variables: **p-values**

Variables	Direct	Indirect	Total
Slope	0.7504	0.1495	0.111999
FCT1	0.7226	0.7669	0.013895
PastT1	0.8229	0.7690	0.862862
PastT5	< 2.22e-16	0.9491	3.36E-05
soyPT1	0.1899	0.0406	0.025073
SoyPT5	0.0013	0.0347	0.000852
GDPcpT1	0.1972	0.0926	0.050845
GDPcpT5	0.0277	0.0137	0.006196
SecMT5	0.1840	0.6983	0.462563

Deforestation model of 2010 (period of 2006—2010)

Coefficients: (asymptotic standard errors)

Variables	Estimate	Std. Error	z value	Pr(>|z|)
(Intercept)	33446000.0000	27432000.0000	12192.0000	0.2228
Slope	211790.0000	2062200.0000	0.1027	0.9182
FCT1	58.7750	14.2830	41150.0000	0.0387
PastT1	-4.4767	45.8380	-0.0977	0.9222
PastT5	3418.3000	442.9100	77177.0000	0.0000
SoyPT5	304.1100	110.9400	27411.0000	0.0061
GDPcpT1	-602580.0000	419560.0000	-14362.0000	0.1509
GDPcpT5	188840.0000	417950.0000	0.4518	0.6514
SecMT5	-80.5530	77.5070	-10393.0000	0.2987
lag.Slope	-2643900.0000	2864800.0000	-0.9229	0.3561
lag.FCT1	24.7150	26.7390	0.9243	0.3553
lag.PastT1	-54.9340	94.4140	-0.5818	0.5607
lag.PastT5	-1027.0000	1005.8000	-10211.0000	0.3072
lag.SoyPT5	265.0100	227.7400	11637.0000	0.2446
lag.GDPcpT1	176810.0000	959050.0000	0.1844	0.8537
lag.GDPcpT5	831210.0000	1084900.0000	0.7662	0.4436
lag.SecMT5	48.9390	157.0900	0.3115	0.7554

Significance of the impact of variables: **p-values**

Variables	Direct	Indirect	Total
Slope	0.9182	0.3561	0.2911
FCT1	0.3872	0.3553	0.0027
PastT1	0.9222	0.5607	0.5704
PastT5	0.0000	0.3072	0.0298
SoyPT5	0.0061	0.2446	0.0352
GDPcpT1	0.1509	0.8537	0.6677
GDPcpT5	0.6514	0.4436	0.4347
SecMT5	0.2987	0.7554	0.8622

Deforestation model of 2011 (period of 2007—2011)

Coefficients: (asymptotic standard errors)

Variables	Estimate	Std. Error	z value	Pr(>|z|)
(Intercept)	10150000.0000	19551000.0000	0.5191	0.603661
Slope	730330.0000	1705800.0000	0.4281	0.668553
FCT1	45.7080	12.0400	37962.0000	1.47E-04
PastT1	30.2500	37.2940	0.8111	0.417293
PastT5	4722.0000	464.9900	101552.0000	< 2.2e-16
soyPT1	-79.4790	31.1180	-25541.0000	0.010645
SoyPT5	615.4900	100.0500	61519.0000	7.65E-07
GDPcpT1	381550.0000	275050.0000	13872.0000	0.165383
GDPcpT5	738860.0000	768220.0000	0.9618	0.336159
SecMT5	-116.2200	101.8600	-11409.0000	0.253902
lag.Slope	-1698200.0000	2152500.0000	-0.7889	0.430165
lag.FCT1	6.5984	19.5370	0.3377	0.735562
lag.PastT1	-88.4350	69.4240	-12738.0000	0.202721
lag.PastT5	498.3300	890.3100	0.5597	0.575668
lag.soyPT1	-235.2500	69.8830	-33664.0000	0.000762
lag.SoyPT5	819.1400	220.4300	37160.0000	0.000202
lag.GDPcpT1	879760.0000	673880.0000	13055.0000	0.191717
lag.GDPcpT5	-1042800.0000	1864000.0000	-0.5595	0.575848
lag.SecMT5	-220.5100	222.5600	-0.9908	0.321777

Significance of the impact of variables: **p-values**

Variables	Direct	Indirect	Total
Slope	0.6686	0.4302	0.503807
FCT1	0.0001	0.7356	0.00269
PastT1	0.4173	0.2027	0.39603
PastT5	< 2.22e-16	0.5757	2.29E-05
soyPT1	0.0106	0.0008	3.89E-01
SoyPT5	0.0000	0.0002	2.81E-05
GDPcpT1	0.1654	0.1917	0.078491
GDPcpT5	0.3362	0.5758	0.881735
SecMT5	0.2539	0.3218	0.167656

Deforestation model of 2012 (period of 2008—2012)

Coefficients: (asymptotic standard errors)

Variables	Estimate	Std. Error	z value	Pr(>|z|)
(Intercept)	-23797000.0000	27293000.0000	-0.8719	0.3833
Slope	-3176600.0000	1709400.0000	-18583.0000	0.0631
FCT1	43.6100	12.2110	35714.0000	0.0004
PastT1	114.6900	38.5420	29758.0000	0.0029
PastT5	3208.9000	411.1500	78046.0000	0.0000
SoyPT5	642.7000	130.5500	49230.0000	0.0009
GDPcpT1	-80433.0000	241000.0000	-0.3338	0.7386
GDPcpT5	167480.0000	471920.0000	0.3549	0.7227
SecMT5	-91.3010	44.3780	-20573.0000	0.0397
lag.Slope	4642400.0000	2470500.0000	18792.0000	0.0602
lag.FCT1	5.7178	21.7450	0.2629	0.7926
lag.PastT1	-86.4580	81.1910	-10649.0000	0.2869
lag.PastT5	22.0090	857.2400	0.0257	0.9795
lag.SoyPT5	374.9300	300.8500	12462.0000	0.2127
lag.GDPcpT1	474760.0000	622610.0000	0.7625	0.4457
lag.GDPcpT5	686240.0000	1198100.0000	0.5728	0.5668
lag.SecMT5	61.8300	88.9130	0.6954	0.4868

Significance of the impact of variables: **p-values**

Variables	Direct	Indirect	Total
Slope	0.0631	0.0602	0.4509
FCT1	0.0004	0.7926	0.0321
PastT1	0.0029	0.2869	0.7505
PastT5	0.0000	0.9795	0.0009
SoyPT5	0.0085	0.2127	0.0027
GDPcpT1	0.7386	0.4457	0.5681
GDPcpT5	0.7227	0.5668	0.5558
SecMT5	0.0397	0.4868	0.7674

Deforestation model of 2013 (period of 2009—2013)

Coefficients: (asymptotic standard errors)

Variables	Estimate	Std. Error	z value	Pr(>|z|)
(Intercept)	959100.0000	26752000.0000	0.0359	0.9714
Slope	-5048700.0000	2061900.0000	-24486.0000	0.0143
FCT1	66.6070	14.7310	45216.0000	0.0061
PastT1	77.1120	47.4750	16243.0000	0.1043
PastT5	3000.1000	383.1900	78294.0000	0.0000
SoyPT5	667.9000	130.7800	51069.0000	0.0003
GDPcpT1	-493430.0000	316810.0000	-15575.0000	0.1194
GDPcpT5	162660.0000	468520.0000	0.3472	0.7285
SecMT5	5.4664	21.4300	0.2551	0.7987
lag.Slope	6615200.0000	2749500.0000	24060.0000	0.0161
lag.FCT1	24.1660	24.2440	0.9968	0.3189
lag.PastT1	-195.9100	90.9220	-21547.0000	0.0312
lag.PastT5	316.2200	734.5100	0.4305	0.6668
lag.SoyPT5	879.8800	289.8100	30361.0000	0.0024
lag.GDPcpT1	-591460.0000	766960.0000	-0.7712	0.4406
lag.GDPcpT5	-88244.0000	1223000.0000	-0.0722	0.9425
lag.SecMT5	-13.9040	45.0440	-0.3087	0.7576

Significance of the impact of variables: **p-values**

Variables	Direct	Indirect	Total
Slope	0.0143	0.0161	0.4047
FCT1	0.0614	0.3189	0.5899
PastT1	0.1043	0.0312	0.1989
PastT5	0.0000	0.6668	0.6083
SoyPT5	0.0033	0.0024	0.0392
GDPcpT1	0.1194	0.4406	0.1871
GDPcpT5	0.7285	0.9425	0.9586
SecMT5	0.7987	0.7576	0.8740

Deforestation model of 2014 (period of 2010—2014)

Coefficients: (asymptotic standard errors)

Variables	Estimate	Std. Error	z value	Pr(>|z|)
(Intercept)	5457800.0000	34953000.0000	0.1561	0.8759
Slope	-7980500.0000	2419500.0000	-32984.0000	0.0010
FCT1	67.5580	17.1210	39460.0000	0.0795
PastT1	151.5800	57.0610	26565.0000	0.0079
PastT5	2617.6000	389.5400	67198.0000	0.0000
soyPT1	-44.6390	52.3500	-0.8527	0.3938
SoyPT5	694.8800	132.7700	52338.0000	0.0002
GDPcpT1	-169410.0000	389420.0000	-0.4350	0.6635
GDPcpT5	-44508.0000	379440.0000	-0.1173	0.9066
SecMT5	-12.2100	100.9100	-0.1210	0.9037
lag.Slope	10706000.0000	3323400.0000	32213.0000	0.0013
lag.FCT1	73.1700	30.7060	23829.0000	0.0172
lag.PastT1	-289.4800	118.4400	-24442.0000	0.0145
lag.PastT5	-1753.6000	823.8300	-21286.0000	0.0333
lag.soyPT1	-79.1610	119.9900	-0.6597	0.5094
lag.SoyPT5	-94.1220	303.6000	-0.3100	0.7565
lag.GDPcpT1	-765430.0000	961330.0000	-0.7962	0.4259
lag.GDPcpT5	886350.0000	1112800.0000	0.7965	0.4257
lag.SecMT5	172.5700	233.1900	0.7400	0.4593

Significance of the impact of variables: **p-values**

Variables	Direct	Indirect	Total
Slope	0.0010	0.0013	0.2718
FCT1	0.7945	0.0172	0.0557
PastT1	0.0079	0.0145	0.2726
PastT5	0.0000	0.0333	0.3478
soyPT1	0.3938	0.5094	0.3148
SoyPT5	0.0017	0.7565	0.0825
GDPcpT1	0.6635	0.4259	0.4047
GDPcpT5	0.9066	0.4257	0.5295
SecMT5	0.9037	0.4593	0.5398

Deforestation model of 2015 (period of 2011—2015)

Coefficients: (asymptotic standard errors)

Variables	Estimate	Std. Error	z value	Pr(>|z|)
(Intercept)	21311000.0000	47439000.0000	0.4492	0.6533
Slope	-3524400.0000	2682800.0000	-13137.0000	0.1889
FCT1	82.5900	19.1720	43079.0000	0.0165
PastT1	51.2090	65.1870	0.7856	0.4321
PastT5	2724.4000	387.7700	70259.0000	0.0000
SoyPT5	800.5200	115.8400	69107.0000	0.0000
GDPcpT1	-573760.0000	428990.0000	-13375.0000	0.1811
GDPcpT5	359930.0000	698520.0000	0.5153	0.6064
SecMT5	54.5240	39.6100	13765.0000	0.1687
lag.Slope	5525300.0000	3877600.0000	14249.0000	0.1542
lag.FCT1	70.7300	34.1320	20723.0000	0.0382
lag.PastT1	-203.4000	132.3700	-15367.0000	0.1244
lag.PastT5	-3530.4000	930.4700	-37942.0000	0.0001
lag.SoyPT5	-577.8600	280.3600	-20611.0000	0.0393
lag.GDPcpT1	-191720.0000	1050300.0000	-0.1825	0.8552
lag.GDPcpT5	359230.0000	1995000.0000	0.1801	0.8571
lag.SecMT5	-47.5650	86.2030	-0.5518	0.5811

Significance of the impact of variables: **p-values**

Variables	Direct	Indirect	Total
Slope	0.1889	0.1542	0.4908
FCT1	0.1648	0.0382	0.1149
PastT1	0.4321	0.1244	0.2889
PastT5	0.0000	0.0001	0.4292
SoyPT5	0.0000	0.0393	0.4712
GDPcpT1	0.1811	0.8552	0.5230
GDPcpT5	0.6064	0.8571	0.7629
SecMT5	0.1687	0.5811	0.9466

Deforestation model of 2016 (period of 2012—2016)

Coefficients: (asymptotic standard errors)

Variables	Estimate	Std. Error	z value	Pr(>|z|)
(Intercept)	34653000.0000	37448000.0000	0.9254	0.3548
Slope	-4424300.0000	2803800.0000	-15780.0000	0.1146
FCT1	87.6970	20.2240	43363.0000	0.0145
PastT1	159.5400	64.9000	24582.0000	0.0140
PastT5	2331.7000	361.9900	64414.0000	0.0000
soyPT1	15.6750	34.6430	0.4525	0.6509
SoyPT5	360.6000	118.7200	30374.0000	0.0024
GDPcpT1	-38694.0000	370560.0000	-0.1044	0.9168
GDPcpT5	-128460.0000	816920.0000	-0.1572	0.8751
SecMT5	26.7490	126.7200	0.2111	0.8328
lag.Slope	4508900.0000	3637200.0000	12397.0000	0.2151
lag.FCT1	72.3500	34.6470	20882.0000	0.0368
lag.PastT1	-317.1900	127.9200	-24796.0000	0.0132
lag.PastT5	-1980.4000	792.0200	-25005.0000	0.0124
lag.soyPT1	-40.0480	73.4110	-0.5455	0.5854
lag.SoyPT5	284.2900	351.3900	0.8091	0.4185
lag.GDPcpT1	-259950.0000	1019500.0000	-0.2550	0.7987
lag.GDPcpT5	958770.0000	2211600.0000	0.4335	0.6646
lag.SecMT5	-525.5000	237.2300	-22151.0000	0.0268

Significance of the impact of variables: **p-values**

Variables	Direct	Indirect	Total
Slope	0.1146	0.2151	0.9735
FCT1	0.1449	0.0368	0.0000
PastT1	0.0140	0.0132	0.2351
PastT5	0.0000	0.0124	0.6793
soyPT1	0.6509	0.5854	0.7679
SoyPT5	0.0024	0.4185	0.1172
GDPcpT1	0.9168	0.7987	0.7967
GDPcpT5	0.8751	0.6646	0.7518
SecMT5	0.8328	0.0268	0.0469

Deforestation model of 2017 (period of 2013—2017)

Coefficients: (asymptotic standard errors)

Variables	Estimate	Std. Error	z value	Pr(>|z|)
(Intercept)	6365500.0000	37030000.0000	0.1719	0.8635
Slope	-2372800.0000	2695900.0000	-0.8802	0.3788
FCT1	72.2100	20.5130	35202.0000	0.0004
PastT1	201.6300	62.8810	32064.0000	0.0013
PastT5	3899.8000	438.5500	88924.0000	< 2.2e-16
soyPT1	-6.0416	45.8170	-0.1319	0.8951
SoyPT5	459.2200	113.1700	40579.0000	0.0495
GDPcpT1	243210.0000	349570.0000	0.6957	0.4866
GDPcpT5	197550.0000	700340.0000	0.2821	0.7779
SecMT5	-80.4110	75.3980	-10665.0000	0.2862
lag.Slope	2837600.0000	3403000.0000	0.8339	0.4044
lag.FCT1	55.1310	32.5620	16931.0000	0.0904
lag.PastT1	-251.7900	118.3500	-21276.0000	0.0334
lag.PastT5	-763.7300	840.3100	-0.9089	0.3634
lag.soyPT1	63.2810	90.6760	0.6979	0.4853
lag.SoyPT5	390.0900	223.0500	17489.0000	0.0803
lag.GDPcpT1	-152750.0000	902730.0000	-0.1692	0.8656
lag.GDPcpT5	1865200.0000	1844600.0000	10112.0000	0.3119
lag.SecMT5	-333.1500	148.3800	-22453.0000	0.0247

Significance of the impact of variables: **p-values**

Variables	Direct	Indirect	Total
Slope	0.3788	0.4044	0.8329
FCT1	0.0004	0.0904	0.0318
PastT1	0.0013	0.0334	0.6600
PastT5	< 2.22e-16	0.3634	0.0006
soyPT1	0.8951	0.4853	0.5571
SoyPT5	0.4951	0.0803	0.0006
GDPcpT1	0.4866	0.8656	0.9292
GDPcpT5	0.7779	0.3119	0.3208
SecMT5	0.2862	0.0247	0.0120

Deforestation model of 2018 (period of 2014—2018)

Coefficients: (asymptotic standard errors)

Variables	Estimate	Std. Error	z value	Pr(>|z|)
(Intercept)	8636000.0000	35898000.0000	0.2406	0.8099
Slope	-1498000.0000	2444700.0000	-0.6128	0.5400
FCT1	103.7400	18.3410	56562.0000	0.0000
PastT1	193.1200	58.2190	33171.0000	0.0009
PastT5	3905.4000	427.3000	91398.0000	< 2.2e-16
soyPT1	-18.3400	34.9590	-0.5246	0.5999
SoyPT5	278.2200	144.4700	19258.0000	0.0541
GDPcpT1	82536.0000	298790.0000	0.2762	0.7824
GDPcpT5	29493.0000	433720.0000	0.0680	0.9458
SecMT5	62.4860	71.4130	0.8750	0.3816
lag.Slope	2243500.0000	3083500.0000	0.7276	0.4669
lag.FCT1	25.6680	29.8760	0.8592	0.3903
lag.PastT1	-109.8100	109.0000	-10074.0000	0.3137
lag.PastT5	-160.1600	752.6900	-0.2128	0.8315
lag.soyPT1	15.8230	75.4710	0.2097	0.8339
lag.SoyPT5	281.7300	302.0900	0.9326	0.3510
lag.GDPcpT1	-506430.0000	806490.0000	-0.6279	0.5300
lag.GDPcpT5	1796500.0000	1168600.0000	15373.0000	0.1242
lag.SecMT5	-188.3200	137.3700	-13709.0000	0.1704

Significance of the impact of variables: **p-values**

Variables	Direct	Indirect	Total
Slope	0.5400	0.4669	0.7191
FCT1	0.0002	0.3903	0.0051
PastT1	0.0009	0.3137	0.4242
PastT5	< 2.22e-16	0.8315	0.0152
soyPT1	0.5999	0.8339	0.9762
SoyPT5	0.0541	0.3510	0.0899
GDPcpT1	0.7824	0.5300	0.6461
GDPcpT5	0.9458	0.1242	0.1573
SecMT5	0.3816	0.1704	0.3831

SUPPLEMENTARY INFORMATION 2

GDP per capita models. Results from the Spatial Durbin Error Model (SDEM)

For a better understand of each model's subset result, we firstly present a list with the full names and abbreviations of each variable as follow:

FCT1 = natural forest cover at the first year of the model subset simulation (e.g., FC of 2000, model subset of 2000—2004)

Def = deforestation in the period of the model subset

PastT1 = pastureland area at the first year of the model subset simulation

PastT5 = change in pastureland area over the five-year period ($\Delta t5$)

SoyPT1 = soybean production at the first year of the model subset simulation

SoyPT5 = change in soybean production over the five-year period ($\Delta t5$)

GDPcpT1 = GDP per capita at the first year of the model subset simulation

GDPcpT5 = change in GDP per capita over the five-year period ($\Delta t5$)

SecMT5 = maize second-crop production change over the five-year period ($\Delta t5$)

LaborAgT1 = number of agricultural laborers at the first year of the model subset simulation

LaborAgT5 = change in the number of agricultural laborers over the five-year period ($\Delta t5$)

CattleT1 = number of animals at the first year of the model subset simulation

CattleT5 = change in the number of animals over the five-year period ($\Delta t5$)

All variables with "lag." represent the indirect (spillover) effect of the respective variable on its neighbors.

GDP per capita model of 2004 (period of 2000—2004)

Coefficients: (asymptotic standard errors)

Variables	Estimate	Std. Error	z value	Pr(>|z|)
(Intercept)	9921.8000	31199.0000	0.3180	0.7505
FCT1	0.0033	0.0372	0.0893	0.9288
Def	-0.8193	0.5887	-13918.0000	0.1640
PastT1	0.1844	0.2468	0.7472	0.4550
soyPT1	-0.0285	0.1132	-0.2519	0.8011
SoyPT5	1.3595	0.1818	74801.0000	0.0000
SecMT5	0.3490	0.4336	0.8049	0.4209
LaborAgT1	-6.2304	31.3900	-0.1985	0.8427
LaborAgT5	-17.9730	37.3100	-0.4817	0.6300
CattleT1	-0.2717	0.1840	-14772.0000	0.1396
CattleT5	0.3302	0.2581	12793.0000	0.2008
lag.FCT1	0.0414	0.0596	0.6945	0.4874
lag.Def	-0.7962	1.0429	-0.7634	0.4452
lag.PastT1	0.7256	0.3037	23893.0000	0.0169
lag.soyPT1	0.5309	0.2806	18919.0000	0.0585
lag.SoyPT5	-0.7189	0.4107	-17506.0000	0.0800
lag.SecMT5	3.7614	1.4005	26858.0000	0.0072
lag.LaborAgT1	57.7320	84.6120	0.6823	0.4950
lag.LaborAgT5	6.7930	122.2500	0.0556	0.9557
lag.CattleT1	-0.6317	0.3166	-19950.0000	0.0460
lag.CattleT5	0.4961	0.4745	10455.0000	0.2958

Significance of the impact of variables: **p-values**

Variables	Direct	Indirect	Total
FCT1	0.9288	0.4874	0.3508
Def	0.1640	0.4452	0.0921
PastT1	0.4550	0.0169	0.0010
soyPT1	0.8011	0.0585	0.0708
SoyPT5	0.0000	0.0800	0.1771
SecMT5	0.4209	0.0072	0.0042
LaborAgT1	0.8427	0.4950	0.5318
LaborAgT5	0.6300	0.9557	0.9322
CattleT1	0.1396	0.0460	0.0068
CattleT5	0.2008	0.2958	0.0678

GDP per capita model of 2005 (period of 2001—2005)

Coefficients: (asymptotic standard errors)

Variables	Estimate	Std. Error	z value	Pr(>|z|)
(Intercept)	24300.0000	36113.0000	0.6729	0.5010
FCT1	-0.0137	0.0268	-0.5097	0.6103
Def	-0.3312	0.3936	-0.8415	0.4001
PastT1	0.1512	0.1777	0.8510	0.3948
soyPT1	0.0599	0.0796	0.7526	0.4517
SoyPT5	0.2401	0.1414	16980.0000	0.0895
SecMT5	1.4179	0.3955	35854.0000	0.0003
LaborAgT1	-17.5530	26.0580	-0.6736	0.5005
LaborAgT5	100.6600	53.4530	18831.0000	0.0597
CattleT1	-0.2308	0.1432	-16117.0000	0.1070
CattleT5	0.1149	0.1897	0.6056	0.5448
lag.FCT1	-0.0131	0.0530	-0.2481	0.8040
lag.Def	0.1772	0.8627	0.2054	0.8373
lag.PastT1	0.7578	0.2960	25604.0000	0.0105
lag.soyPT1	0.1800	0.2198	0.8188	0.4129
lag.SoyPT5	-0.1550	0.3617	-0.4285	0.6683
lag.SecMT5	-0.7628	1.1294	-0.6754	0.4994
lag.LaborAgT1	45.0810	65.8480	0.6846	0.4936
lag.LaborAgT5	88.3670	135.8600	0.6504	0.5154
lag.CattleT1	-0.6638	0.2722	-24386.0000	0.0147
lag.CattleT5	0.3734	0.4410	0.8468	0.3971

Significance of the impact of variables: **p-values**

Variables	Direct	Indirect	Total
FCT1	0.6103	0.8040	0.6450
Def	0.4001	0.8373	0.8729
PastT1	0.3948	0.0105	0.0135
soyPT1	0.4517	0.4129	0.3693
SoyPT5	0.0895	0.6683	0.8437
SecMT5	0.0003	0.4994	0.6063
LaborAgT1	0.5005	0.4936	0.7211
LaborAgT5	0.0597	0.5154	0.2590
CattleT1	0.1070	0.0147	0.0073
CattleT5	0.5448	0.3971	0.3445

GDP per capita model of 2006 (period of 2002—2006)

Coefficients: (asymptotic standard errors)

Variables	Estimate	Std. Error	z value	Pr(>|z|)
(Intercept)	61643.0000	25572.0000	24106.0000	0.0159
FCT1	-0.0064	0.0143	-0.4478	0.6543
PastT1	-0.0110	0.0635	-0.1732	0.8625
soyPT1	-0.0933	0.0418	-22318.0000	0.0256
SoyPT5	0.0643	0.0950	0.6774	0.4982
SecMT5	0.6176	0.2418	25546.0000	0.0106
LaborAgT1	-0.1901	14.7390	-0.0129	0.9897
LaborAgT5	32.6360	30.2580	10786.0000	0.2808
CattleT5	-0.0020	0.1306	-0.0150	0.9881
lag.FCT1	0.0094	0.0294	0.3182	0.7503
lag.PastT1	-0.0667	0.1351	-0.4938	0.6215
lag.soyPT1	0.1249	0.0829	15078.0000	0.1316
lag.SoyPT5	-0.2231	0.2384	-0.9360	0.3493
lag.SecMT5	-0.0465	0.7184	-0.0647	0.9484
lag.LaborAgT1	-8.1922	34.9740	-0.2342	0.8148
lag.LaborAgT5	6.4013	73.0060	0.0877	0.9301
lag.CattleT5	-0.2828	0.2794	-10122.0000	0.3115

Significance of the impact of variables: **p-values**

Variables	Direct	Indirect	Total
FCT1	0.6543	0.7503	0.9318
PastT1	0.8625	0.6215	0.6324
soyPT1	0.0256	0.1316	0.7493
SoyPT5	0.4982	0.3493	0.5863
SecMT5	0.0106	0.9484	0.5100
LaborAgT1	0.9897	0.8148	0.8432
LaborAgT5	0.2808	0.9301	0.6568
CattleT5	0.9881	0.3115	0.3996

GDP per capita model of 2007 (period of 2003—2007)

Coefficients: (asymptotic standard errors)

Variables	Estimate	Std. Error	z value	Pr(>|z|)
(Intercept)	21764.0000	17008.0000	12796.0000	0.2007
FCT1	0.0188	0.0176	10646.0000	0.2871
Def	-0.0643	0.3649	-0.1761	0.8602
PastT1	0.0336	0.0502	0.6702	0.5027
PastT5	-0.5709	0.3493	-16342.0000	0.1022
soyPT1	-0.1980	0.0386	-51349.0000	0.0003
SoyPT5	-0.1399	0.1058	-13221.0000	0.1861
SecMT5	0.7489	0.2100	35657.0000	0.0004
LaborAgT1	6.9054	11.0960	0.6223	0.5337
LaborAgT5	-26.7280	17.5330	-15244.0000	0.1274
CattleT5	0.2374	0.1573	15099.0000	0.1311
lag.FCT1	-0.0521	0.0319	-16357.0000	0.1019
lag.Def	3.2852	0.7969	41226.0000	0.0375
lag.PastT1	-0.1285	0.0907	-14164.0000	0.1567
lag.PastT5	-2.1303	0.7949	-26800.0000	0.0074
lag.soyPT1	-0.0617	0.0832	-0.7421	0.4580
lag.SoyPT5	-0.6873	0.2945	-23336.0000	0.0196
lag.SecMT5	-0.0539	0.5871	-0.0917	0.9269
lag.LaborAgT1	15.5570	22.8190	0.6818	0.4954
lag.LaborAgT5	42.6930	41.9870	10168.0000	0.3092
lag.CattleT5	-0.0930	0.3115	-0.2987	0.7652

Significance of the impact of variables: **p-values**

Variables	Direct	Indirect	Total
FCT1	0.2871	0.1019	0.2713
Def	0.8602	0.3746	0.0001
PastT1	0.5027	0.1567	0.3029
PastT5	0.1022	0.0074	0.0020
soyPT1	0.0028	0.4580	0.0020
SoyPT5	0.1861	0.0196	0.0131
SecMT5	0.0004	0.9269	0.3061
LaborAgT1	0.5337	0.4954	0.3457
LaborAgT5	0.1274	0.3092	0.7171
CattleT5	0.1311	0.7652	0.6676

GDP per capita model of 2008 (period of 2004—2008)

Coefficients: (asymptotic standard errors)

Variables	Estimate	Std. Error	z value	Pr(>|z|)
(Intercept)	55060.0000	14596.0000	37722.0000	0.0002
FCT1	-0.0244	0.0168	-14488.0000	0.1474
PastT1	0.0311	0.0557	0.5591	0.5761
PastT5	-0.1918	0.3461	-0.5543	0.5794
soyPT1	-0.3809	0.0473	-80451.0000	0.0000
SoyPT5	0.6259	0.0812	77082.0000	0.0000
SecMT5	0.9589	0.1115	86011.0000	< 2.2e-16
LaborAgT1	41.7660	11.9390	34981.0000	0.0005
LaborAgT5	57.6310	18.3270	31445.0000	0.0017
CattleT5	0.2485	0.2049	12128.0000	0.2252
lag.FCT1	0.0442	0.0265	16677.0000	0.0954
lag.PastT1	-0.1039	0.0849	-12230.0000	0.2213
lag.PastT5	-1.0925	0.6499	-16809.0000	0.0928
lag.soyPT1	-0.0861	0.0919	-0.9373	0.3486
lag.SoyPT5	-0.1877	0.1595	-11768.0000	0.2393
lag.SecMT5	0.2720	0.2972	0.9150	0.3602
lag.LaborAgT1	-35.9930	21.2200	-16962.0000	0.0898
lag.LaborAgT5	-67.9420	48.5510	-13994.0000	0.1617
lag.CattleT5	0.2048	0.3287	0.6231	0.5332

Significance of the impact of variables: **p-values**

Variables	Direct	Indirect	Total
FCT1	0.1474	0.0954	0.3617
PastT1	0.5761	0.2213	0.3244
PastT5	0.5794	0.0928	0.0325
soyPT1	0.0000	0.3486	0.0309
SoyPT5	0.0000	0.2393	0.0177
SecMT5	< 2.22e-16	0.3602	0.0002
LaborAgT1	0.0005	0.0898	0.7700
LaborAgT5	0.0017	0.1617	0.8232
CattleT5	0.2252	0.5332	0.0860

GDP per capita model of 2009 (period of 2005—2009)

Coefficients: (asymptotic standard errors)

Variables	Estimate	Std. Error	z value	Pr(>|z|)
(Intercept)	56928.0000	13608.0000	41836.0000	0.0000
FCT1	-0.0006	0.0171	-0.0357	0.9715
PastT1	0.0874	0.0568	15381.0000	0.1240
PastT5	-0.8198	0.4108	-19957.0000	0.0460
soyPT1	0.0752	0.0341	22071.0000	0.0273
SoyPT5	0.2540	0.1354	18766.0000	0.0606
SecMT5	0.0095	0.0487	0.1945	0.8458
LaborAgT1	-11.3100	12.4100	-0.9113	0.3621
LaborAgT5	20.2210	17.9990	11235.0000	0.2612
CattleT5	0.1730	0.1741	0.9936	0.3204
lag.FCT1	0.0463	0.0240	19323.0000	0.0533
lag.PastT1	-0.2307	0.0842	-27416.0000	0.0061
lag.PastT5	0.2867	0.6952	0.4124	0.6800
lag.soyPT1	0.0548	0.0529	10350.0000	0.3006
lag.SoyPT5	0.2609	0.2538	10278.0000	0.3040
lag.SecMT5	-0.1485	0.1257	-11811.0000	0.2375
lag.LaborAgT1	-40.2130	19.0120	-21152.0000	0.0344
lag.LaborAgT5	72.0130	42.9040	16784.0000	0.0933
lag.CattleT5	-0.2397	0.2518	-0.9519	0.3412

Significance of the impact of variables: **p-values**

Variables	Direct	Indirect	Total
FCT1	0.9715	0.0533	0.0057
PastT1	0.1240	0.0061	0.0277
PastT5	0.0460	0.6800	0.3569
soyPT1	0.0273	0.3006	0.0046
SoyPT5	0.0606	0.3040	0.0160
SecMT5	0.8458	0.2375	0.2977
LaborAgT1	0.3621	0.0344	0.0007
LaborAgT5	0.2612	0.0933	0.0233
CattleT5	0.3204	0.3412	0.7428

GDP per capita model of 2010 (period of 2006—2010)

Coefficients: (asymptotic standard errors)

Variables	Estimate	Std. Error	z value P	P r(>|z|)
(Intercept)	86639.0000	30533.0000	28376.0000	0.0045
FCT1	0.0338	0.0321	10540.0000	0.2919
Def	0.3831	2.1346	0.1795	0.8576
PastT5	-1.1365	1.2250	-0.9277	0.3536
soyPT1	-0.0415	0.1143	-0.3630	0.7166
SoyPT5	0.0842	0.2525	0.3337	0.7386
SecMT5	0.2280	0.3064	0.7441	0.4568
LaborAgT1	-1.7367	25.5230	-0.0680	0.9458
LaborAgT5	-4.7814	34.2680	-0.1395	0.8890
CattleT1	0.0618	0.0767	0.8057	0.4204
CattleT5	0.1884	0.4033	0.4672	0.6404
lag.FCT1	-0.0149	0.0540	-0.2759	0.7826
lag.Def	-2.0971	3.9636	-0.5291	0.5967
lag.PastT5	3.3578	2.1268	15788.0000	0.1144
lag.soyPT1	0.3371	0.1954	17254.0000	0.0845
lag.SoyPT5	-0.1060	0.4524	-0.2344	0.8147
lag.SecMT5	-0.3539	0.5643	-0.6271	0.5306
lag.LaborAgT1	-69.6300	49.9770	-13932.0000	0.1636
lag.LaborAgT5	-39.9360	83.0830	-0.4807	0.6307
lag.CattleT1	0.0343	0.1494	0.2297	0.8183
lag.CattleT5	-0.0631	0.7442	-0.0848	0.9324

Significance of the impact of variables: **p-values**

Variables	Direct	Indirect	Total
FCT1	0.2919	0.7826	0.6889
Def	0.8576	0.5967	0.6559
PastT5	0.3536	0.1144	0.2428
soyPT1	0.7166	0.0845	0.0876
SoyPT5	0.7386	0.8147	0.9626
SecMT5	0.4568	0.5306	0.8111
LaborAgT1	0.9458	0.1636	0.1442
LaborAgT5	0.8890	0.6307	0.6033
CattleT1	0.4204	0.8183	0.4717
CattleT5	0.6404	0.9324	0.8664

GDP per capita model of 2011 (period of 2007—2011)

Coefficients: (asymptotic standard errors)

Variables	Estimate	Std. Error	z value	Pr(>|z|)
(Intercept)	49274.0000	13406.0000	36757.0000	0.0002
FCT1	0.0030	0.0131	0.2276	0.8199
Def	1.8908	0.9116	20742.0000	0.0381
PastT5	-1.0580	0.6756	-15660.0000	0.1173
soyPT1	-0.1527	0.0392	-38918.0000	0.0995
SoyPT5	0.1067	0.1184	0.9012	0.3675
SecMT5	0.2514	0.1146	21940.0000	0.0282
LaborAgT1	40.5190	9.7976	41356.0000	0.0354
LaborAgT5	-63.1460	15.4850	-40780.0000	0.0454
CattleT1	-0.0174	0.0371	-0.4684	0.6395
CattleT5	-0.0358	0.2117	-0.1692	0.8657
lag.FCT1	-0.0163	0.0210	-0.7753	0.4382
lag.Def	0.5158	2.1744	0.2372	0.8125
lag.PastT5	0.5353	1.2883	0.4155	0.6778
lag.soyPT1	-0.0897	0.0718	-12486.0000	0.2118
lag.SoyPT5	0.2526	0.2927	0.8629	0.3882
lag.SecMT5	-0.3365	0.2326	-14470.0000	0.1479
lag.LaborAgT1	-1.4215	21.1410	-0.0672	0.9464
lag.LaborAgT5	5.3348	37.7410	0.1414	0.8876
lag.CattleT1	-0.0407	0.0751	-0.5425	0.5875
lag.CattleT5	0.0996	0.4304	0.2314	0.8170

Significance of the impact of variables: **p-values**

Variables	Direct	Indirect	Total
FCT1	0.8199	0.4382	0.4596
Def	0.0381	0.8125	0.3076
PastT5	0.1173	0.6778	0.6836
soyPT1	0.9949	0.2118	0.0004
SoyPT5	0.3675	0.3882	0.2595
SecMT5	0.0282	0.1479	0.7174
LaborAgT1	0.3540	0.9464	0.0577
LaborAgT5	0.4542	0.8876	0.1633
CattleT1	0.6395	0.5875	0.4316
CattleT5	0.8657	0.8170	0.8919

GDP per capita model of 2012 (period of 2008—2012)

Coefficients: (asymptotic standard errors)

Variables	Estimate	Std. Error	z value	Pr(>|z|)
(Intercept)	46723.0000	23139.0000	20193.0000	0.0435
FCT1	-0.0041	0.0237	-0.1745	0.8615
Def	0.0437	1.5482	0.0282	0.9775
PastT5	-0.0794	0.9102	-0.0872	0.9305
SoyPT5	0.6036	0.2605	23171.0000	0.0205
SecMT5	0.1134	0.0896	12650.0000	0.2059
LaborAgT1	-10.5170	14.6830	-0.7163	0.4738
LaborAgT5	-56.5880	28.5640	-19811.0000	0.0476
CattleT1	-0.0165	0.0609	-0.2705	0.7868
CattleT5	0.3174	0.3118	10182.0000	0.3086
lag.FCT1	0.0179	0.0392	0.4557	0.6486
lag.Def	0.0823	3.0713	0.0268	0.9786
lag.PastT5	0.8313	1.6074	0.5171	0.6051
lag.SoyPT5	0.0091	0.4970	0.0182	0.9854
lag.SecMT5	-0.1681	0.1583	-10615.0000	0.2885
lag.LaborAgT1	40.4480	31.0950	13008.0000	0.1933
lag.LaborAgT5	-42.8270	63.0590	-0.6792	0.4970
lag.CattleT1	0.0040	0.1171	0.0345	0.9724
lag.CattleT5	-0.6540	0.6098	-10725.0000	0.2835

Significance of the impact of variables: **p-values**

Variables	Direct	Indirect	Total
FCT1	0.8615	0.6486	0.7025
Def	0.9775	0.9786	0.9668
PastT5	0.9305	0.6051	0.6428
SoyPT5	0.0205	0.9854	0.2381
SecMT5	0.2059	0.2885	0.7019
LaborAgT1	0.4738	0.1933	0.3251
LaborAgT5	0.0476	0.4970	0.1375
CattleT1	0.7868	0.9724	0.9098
CattleT5	0.3086	0.2835	0.6119

GDP per capita model of 2013 (period of 2009—2013)

Coefficients: (asymptotic standard errors)

Variables	Estimate	Std. Error	z value	Pr(>|z|)
(Intercept)	56471.0000	25157.0000	22447.0000	0.0248
FCT1	-0.0306	0.0292	-10507.0000	0.2934
Def	0.3940	1.5450	0.2550	0.7987
PastT5	-0.8665	0.8234	-10523.0000	0.2927
SoyPT5	0.4875	0.2555	19077.0000	0.0564
SecMT5	-0.0353	0.0420	-0.8416	0.4000
LaborAgT1	5.6568	16.0200	0.3531	0.7240
LaborAgT5	-7.4445	45.5230	-0.1635	0.8701
CattleT1	-0.0428	0.0667	-0.6418	0.5210
CattleT5	0.4307	0.3382	12735.0000	0.2029
lag.FCT1	0.0386	0.0433	0.8906	0.3731
lag.Def	-4.3634	3.1934	-13664.0000	0.1718
lag.PastT5	1.4507	1.4727	0.9851	0.3246
lag.SoyPT5	-0.1093	0.5781	-0.1891	0.8501
lag.SecMT5	0.1276	0.0848	15047.0000	0.1324
lag.LaborAgT1	35.7010	28.2430	12640.0000	0.2062
lag.LaborAgT5	-155.2300	100.5500	-15439.0000	0.1226
lag.CattleT1	0.0853	0.1170	0.7292	0.4659
lag.CattleT5	-0.6632	0.6346	-10452.0000	0.2960

Significance of the impact of variables: **p-values**

Variables	Direct	Indirect	Total
FCT1	0.2934	0.3731	0.8274
Def	0.7987	0.1718	0.2154
PastT5	0.2927	0.3246	0.7056
SoyPT5	0.0564	0.8501	0.5677
SecMT5	0.4000	0.1324	0.2814
LaborAgT1	0.7240	0.2062	0.0979
LaborAgT5	0.8701	0.1226	0.0930
CattleT1	0.5210	0.4659	0.6649
CattleT5	0.2029	0.2960	0.7166

GDP per capita model of 2014 (period of 2010—2014)

Coefficients: (asymptotic standard errors)

Variables	Estimate	Std. Error	z value	Pr(>|z|)
(Intercept)	27432.0000	38872.0000	0.7057	0.4804
FCT1	-0.0604	0.0466	-12948.0000	0.1954
Def	-1.0202	2.1158	-0.4822	0.6297
PastT5	0.1030	1.1550	0.0892	0.9289
SoyPT5	0.7628	0.3564	21402.0000	0.0323
SecMT5	0.1148	0.1526	0.7522	0.4520
LaborAgT1	15.5810	25.8400	0.6030	0.5465
LaborAgT5	-34.7140	67.8800	-0.5114	0.6091
CattleT1	-0.1172	0.1023	-11456.0000	0.2520
CattleT5	0.6606	0.5515	11978.0000	0.2310
lag.FCT1	0.0504	0.0683	0.7375	0.4608
lag.Def	2.0795	4.2327	0.4913	0.6232
lag.PastT5	-0.8370	1.8399	-0.4549	0.6492
lag.SoyPT5	-0.4181	0.6994	-0.5978	0.5500
lag.SecMT5	-0.1533	0.2771	-0.5532	0.5802
lag.LaborAgT1	107.9600	45.8910	23525.0000	0.0187
lag.LaborAgT5	-131.6100	156.5000	-0.8410	0.4004
lag.CattleT1	-0.1126	0.1690	-0.6663	0.5053
lag.CattleT5	-1.4252	0.8243	-17290.0000	0.0838

Significance of the impact of variables: **p-values**

Variables	Direct	Indirect	Total
FCT1	0.1954	0.4608	0.8641
Def	0.6297	0.6232	0.7833
PastT5	0.9289	0.6492	0.6500
SoyPT5	0.0323	0.5500	0.6214
SecMT5	0.4520	0.5802	0.8829
LaborAgT1	0.5465	0.0186	0.0021
LaborAgT5	0.6091	0.4004	0.2715
CattleT1	0.2520	0.5052	0.0958
CattleT5	0.2310	0.0838	0.2944

GDP per capita model of 2015 (period of 2011—2015)

Coefficients: (asymptotic standard errors)

Variables	Estimate	Std. Error	z value	Pr(>|z|)
(Intercept)	75911.0000	25072.0000	30277.0000	0.0025
FCT1	-0.0168	0.0275	-0.6104	0.5416
Def	0.7354	1.1575	0.6353	0.5252
PastT5	-0.5797	0.6562	-0.8834	0.3770
SoyPT5	0.1584	0.1928	0.8214	0.4114
SecMT5	0.0758	0.0582	13020.0000	0.1929
LaborAgT1	-3.5029	14.3620	-0.2439	0.8073
LaborAgT5	2.6932	46.5310	0.0579	0.9538
CattleT1	-0.0168	0.0587	-0.2855	0.7752
CattleT5	-0.0991	0.2879	-0.3441	0.7308
lag.FCT1	0.0029	0.0406	0.0712	0.9432
lag.Def	-2.0883	2.1253	-0.9826	0.3258
lag.PastT5	1.5050	1.3008	11570.0000	0.2473
lag.SoyPT5	0.1070	0.3920	0.2728	0.7850
lag.SecMT5	-0.0820	0.1123	-0.7308	0.4649
lag.LaborAgT1	39.0840	27.0680	14439.0000	0.1488
lag.LaborAgT5	43.3800	111.4800	0.3891	0.6972
lag.CattleT1	-0.0558	0.0999	-0.5586	0.5764
lag.CattleT5	-0.6428	0.4229	-15199.0000	0.1285

Significance of the impact of variables: **p-values**

Variables	Direct	Indirect	Total
FCT1	0.5416	0.9432	0.7122
Def	0.5252	0.3258	0.4985
PastT5	0.3770	0.2473	0.4406
SoyPT5	0.4114	0.7850	0.4764
SecMT5	0.1929	0.4649	0.9561
LaborAgT1	0.8073	0.1488	0.1519
LaborAgT5	0.9538	0.6972	0.7187
CattleT1	0.7752	0.5764	0.4153
CattleT5	0.7308	0.1285	0.0550

GDP per capita model of 2016 (period of 2012—2016)

Coefficients: (asymptotic standard errors)

Variables	Estimate	Std. Error	z value	Pr(>|z|)
(Intercept)	63852.0000	20683.0000	30872.0000	0.0020
FCT1	-0.0122	0.0236	-0.5163	0.6056
Def	-0.7546	0.9177	-0.8223	0.4109
PastT5	0.7374	0.4874	15128.0000	0.1303
soyPT1	0.1371	0.0493	27789.0000	0.0055
SoyPT5	0.5183	0.1209	42871.0000	0.0000
SecMT5	0.1909	0.1384	13796.0000	0.1677
LaborAgT1	4.0404	13.8890	0.2909	0.7711
LaborAgT5	125.7400	55.7980	22535.0000	0.0242
CattleT1	0.0324	0.0536	0.6040	0.5459
CattleT5	-0.3285	0.2441	-13454.0000	0.1785
lag.FCT1	-0.0251	0.0371	-0.6774	0.4982
lag.Def	2.2787	1.9324	11792.0000	0.2383
lag.PastT5	0.0582	1.0017	0.0581	0.9536
lag.soyPT1	0.0185	0.0815	0.2273	0.8202
lag.SoyPT5	0.5670	0.3590	15797.0000	0.1142
lag.SecMT5	0.0731	0.2501	0.2923	0.7700
lag.LaborAgT1	10.3850	29.2510	0.3550	0.7226
lag.LaborAgT5	-255.2000	128.6300	-19840.0000	0.0473
lag.CattleT1	-0.0873	0.0906	-0.9632	0.3354
lag.CattleT5	0.5007	0.3800	13177.0000	0.1876

Significance of the impact of variables: **p-values**

Variables	Direct	Indirect	Total
FCT1	0.6056	0.4982	0.2506
Def	0.4109	0.2383	0.4117
PastT5	0.1303	0.9536	0.3670
soyPT1	0.0055	0.8202	0.0239
SoyPT5	0.1810	0.1142	0.0045
SecMT5	0.1677	0.7700	0.2372
LaborAgT1	0.7711	0.7226	0.5909
LaborAgT5	0.0242	0.0473	0.3280
CattleT1	0.5459	0.3354	0.4612
CattleT5	0.1785	0.1876	0.6383

GDP per capita model of 2017 (period of 2013—2017)

Coefficients: (asymptotic standard errors)

Variables	Estimate	Std. Error	z value	Pr(>|z|)
(Intercept)	40709.0000	22524.0000	18073.0000	0.0707
FCT1	0.0014	0.0255	0.0541	0.9569
Def	0.3109	1.0035	0.3098	0.7567
PastT5	-0.0233	0.6436	-0.0363	0.9711
SoyPT5	0.3243	0.1476	21968.0000	0.0280
SecMT5	0.0614	0.0689	0.8911	0.3729
LaborAgT1	5.7321	12.8700	0.4454	0.6561
LaborAgT5	110.7200	49.7630	22250.0000	0.0261
CattleT1	-0.0470	0.0519	-0.9062	0.3648
CattleT5	-0.0234	0.3383	-0.0691	0.9449
lag.FCT1	-0.0480	0.0426	-11270.0000	0.2597
lag.Def	2.7593	2.2916	12041.0000	0.2286
lag.PastT5	-0.7859	1.3517	-0.5814	0.5609
lag.SoyPT5	0.2164	0.3195	0.6773	0.4982
lag.SecMT5	0.1253	0.1200	10442.0000	0.2964
lag.LaborAgT1	-15.0840	24.5420	-0.6146	0.5388
lag.LaborAgT5	-90.4340	107.1000	-0.8444	0.3984
lag.CattleT1	0.0025	0.0893	0.0274	0.9781
lag.CattleT5	0.8579	0.5774	14859.0000	0.1373

Significance of the impact of variables: **p-values**

Variables	Direct	Indirect	Total
FCT1	0.9569	0.2597	0.2440
Def	0.7567	0.2286	0.1844
PastT5	0.9711	0.5609	0.5379
SoyPT5	0.0280	0.4982	0.1090
SecMT5	0.3729	0.2964	0.1139
LaborAgT1	0.6561	0.5388	0.6694
LaborAgT5	0.0261	0.3984	0.8515
CattleT1	0.3648	0.9781	0.5623
CattleT5	0.9449	0.1373	0.1324

GDP per capita model of 2018 (period of 2014—2018)

Coefficients: (asymptotic standard errors)

Variables	Estimate	Std. Error	z value	Pr(>|z|)
(Intercept)	28716.0000	30590.0000	0.9387	0.3479
FCT1	-0.0139	0.0388	-0.3598	0.7190
Def	-0.3418	1.5525	-0.2202	0.8257
PastT5	0.2328	1.0339	0.2251	0.8219
SoyPT5	0.6280	0.2737	22946.0000	0.0218
SecMT5	-0.0195	0.1195	-0.1631	0.8704
LaborAgT1	6.8381	14.9510	0.4574	0.6474
LaborAgT5	133.7900	47.5200	28156.0000	0.0049
CattleT1	-0.0406	0.0713	-0.5689	0.5694
CattleT5	0.0571	0.3691	0.1548	0.8770
lag.FCT1	-0.0700	0.0695	-10071.0000	0.3139
lag.Def	9.2381	3.1968	28898.0000	0.0039
lag.PastT5	-3.1758	1.8631	-17046.0000	0.0883
lag.SoyPT5	-0.1849	0.4841	-0.3820	0.7025
lag.SecMT5	0.0493	0.1901	0.2591	0.7956
lag.LaborAgT1	-9.9736	27.2270	-0.3663	0.7141
lag.LaborAgT5	56.9620	98.9690	0.5756	0.5649
lag.CattleT1	-0.0924	0.1219	-0.7574	0.4488
lag.CattleT5	-0.4281	0.6575	-0.6511	0.5150

Significance of the impact of variables: **p-values**

Variables	Direct	Indirect	Total
FCT1	0.7190	0.3139	0.2132
Def	0.8257	0.0039	0.0057
PastT5	0.8219	0.0883	0.0808
SoyPT5	0.0218	0.7025	0.3556
SecMT5	0.8704	0.7956	0.8637
LaborAgT1	0.6474	0.7141	0.8968
LaborAgT5	0.0049	0.5649	0.0404
CattleT1	0.5694	0.4488	0.1912
CattleT5	0.8770	0.5150	0.5710

SUPPLEMENTARY INFORMATION 3

GDP per capita models. Results from the Ordinary least square regressions (OLS)

For a better understand of each model's subset result, we firstly present a list with the full names and abbreviations of each variable as follow:

FCT1 = natural forest cover at the first year of the model subset simulation (e.g., FC of 2000, model subset of 2000—2004)

Def = deforestation in the period of the model subset

PastT1 = pastureland area at the first year of the model subset simulation

PastT5 = change in pastureland area over the five-year period ($\Delta t5$)

SoyPT1 = soybean production at the first year of the model subset simulation

SoyPT5 = change in soybean production over the five-year period ($\Delta t5$)

GDPcpT1 = GDP per capita at the first year of the model subset simulation

GDPcpT5 = change in GDP per capita over the five-year period ($\Delta t5$)

SecMT5 = maize second-crop production change over the five-year period ($\Delta t5$)

LaborAgT1 = number of agricultural laborers at the first year of the model subset simulation

LaborAgT5 = change in the number of agricultural laborers over the five-year period ($\Delta t5$)

CattleT1 = number of animals at the first year of the model subset simulation

CattleT5 = change in the number of animals over the five-year period ($\Delta t5$)

GDP OLS model of 2004 (period of 2000—2004)

Coefficients

Variables	Estimate	Std. Error	t value	Pr(>|t|)
(Intercept)	6337.00	1923.00	3296.00	0.00
FCT1	0.00	0.00	0.98	0.33
Def	-0.12	0.05	-2198.00	0.03
PastT1	0.02	0.02	0.86	0.39
soyPT1	-0.01	0.01	-1026.00	0.31
SoyPT5	0.15	0.02	8631.00	0.00
SecMT5	-0.02	0.04	-0.64	0.53
LaborAgT1	1.19	3.17	0.37	0.71
LaborAgT5	1.97	3.99	0.49	0.62
CattleT1	-0.03	0.02	-1528.00	0.13
CattleT5	0.02	0.02	0.75	0.46

GDP OLS model of 2005 (period of 2001—2005)

Coefficients

Variables	Estimate	Std. Error	t value	Pr(>|t|)
(Intercept)	6813.00	1452.00	4692.00	0.00
FCT1	0.00	0.00	-0.03	0.98
Def	-0.03	0.04	-0.74	0.46
PastT1	0.03	0.02	1544.00	0.13
soyPT1	0.01	0.01	1518.00	0.13
SoyPT5	0.02	0.02	1641.00	0.10
SecMT5	0.12	0.04	2715.00	0.01
LaborAgT1	-1.37	2.87	-0.48	0.63
LaborAgT5	8.84	5.76	1534.00	0.13
CattleT1	-0.03	0.01	-2079.00	0.04
CattleT5	0.00	0.02	0.12	0.91

GDP OLS model of 2006 (period of 2002—2006)

Coefficients

Variables	Estimate	Std. Error	t value	Pr(>|t|)
(Intercept)	4558.00	973.80	4681.00	0.00
FCT1	0.00	0.00	-0.27	0.78
PastT1	0.00	0.01	-0.36	0.72
soyPT1	-0.01	0.00	-1387.00	0.17
SoyPT5	0.01	0.01	0.87	0.39
SecMT5	0.05	0.03	1822.00	0.07
LaborAgT1	0.55	1.60	0.34	0.73
LaborAgT5	3.59	3.47	1035.00	0.30
CattleT5	-0.01	0.01	-0.55	0.59

GDP OLS model of 2007 (period of 2003—2007)

Coefficients

Variables	Estimate	Std. Error	t value	Pr(>|t|)
(Intercept)	2358.00	820.20	2875.00	0.00
FCT1	0.00	0.00	0.20	0.84
Def	0.02	0.04	0.65	0.51
PastT1	0.00	0.01	0.18	0.86
PastT5	-0.05	0.04	-1417.00	0.16
soyPT1	-0.02	0.00	-4691.00	0.00
SoyPT5	-0.01	0.01	-0.57	0.57
SecMT5	0.07	0.02	3113.00	0.00
LaborAgT1	0.37	1.17	0.32	0.75
LaborAgT5	-1.21	1.91	-0.63	0.53
CattleT5	0.01	0.02	0.81	0.42

GDP OLS model of 2008 (period of 2004—2008)

Coefficients

Variables	Estimate	Std. Error	t value	Pr(>|t|)
(Intercept)	1670.00	831.80	2007.00	0.05
FCT1	0.00	0.00	-1055.00	0.29
PastT1	0.00	0.01	0.34	0.73
PastT5	-0.03	0.03	-0.96	0.34
soyPT1	-0.04	0.00	-8675.00	0.00
SoyPT5	0.06	0.01	7752.00	0.00
SecMT5	0.09	0.01	7732.00	0.00
LaborAgT1	4.20	1.15	3659.00	0.00
LaborAgT5	5.68	1.89	3001.00	0.00
CattleT5	0.03	0.02	1980.00	0.05

GDP OLS model of 2009 (period of 2005—2009)

Coefficients

Variables	Estimate	Std. Error	t value	Pr(>|t|)
(Intercept)	2878.00	876.10	3286.00	0.00
FCT1	0.00	0.00	1554.00	0.12
PastT1	0.00	0.01	0.76	0.45
PastT5	-0.07	0.04	-1607.00	0.11
soyPT1	0.01	0.00	3401.00	0.00
SoyPT5	0.03	0.01	1949.00	0.05
SecMT5	0.01	0.01	1086.00	0.28
LaborAgT1	-2.08	1.16	-1794.00	0.08
LaborAgT5	2.62	1.92	1360.00	0.18
CattleT5	0.01	0.02	0.82	0.41

GDP OLS model of 2010 (period of 2006—2010)

Coefficients

Variables	Estimate	Std. Error	t value	Pr(>|t|)
(Intercept)	6156.00	1682.00	3661.00	0.000365
FCT1	0.00	0.00	1305.00	0.19
Def	0.01	0.20	0.03	0.98
PastT5	-0.05	0.11	-0.44	0.66
soyPT1	0.00	0.01	0.33	0.74
SoyPT5	0.01	0.03	0.32	0.75
SecMT5	0.02	0.03	0.56	0.57
LaborAgT1	-1.31	2.52	-0.52	0.60
LaborAgT5	-0.97	3.60	-0.27	0.79
CattleT1	0.01	0.01	0.98	0.33
CattleT5	0.02	0.04	0.46	0.64

GDP OLS model of 2011 (period of 2007—2011)

Coefficients

Variables	Estimate	Std. Error	t value	Pr(>|t|)
(Intercept)	3377.00	696.30	4851.00	0.00
FCT1	0.00	0.00	-0.29	0.77
Def	0.23	0.09	2502.00	0.01
PastT5	-0.14	0.06	-2202.00	0.03
soyPT1	-0.02	0.00	-4870.00	0.00
SoyPT5	0.00	0.01	0.06	0.95
SecMT5	0.03	0.01	2848.00	0.01
LaborAgT1	4.12	0.96	4312.00	0.00
LaborAgT5	-6.56	1.64	-4011.00	0.00
CattleT1	0.00	0.00	-0.25	0.80
CattleT5	0.00	0.02	0.03	0.97

GDP OLS model of 2012 (period of 2008—2012)

Coefficients

Variables	Estimate	Std. Error	t value	Pr(>|t|)
(Intercept)	5381.00	1215.00	4429.00	0.00
FCT1	0.00	0.00	-0.25	0.80
Def	-0.05	0.15	-0.35	0.73
PastT5	0.05	0.09	0.62	0.54
SoyPT5	0.06	0.03	2466.00	0.02
SecMT5	0.00	0.01	0.59	0.56
LaborAgT1	0.16	1.41	0.11	0.91
LaborAgT5	-5.90	2.95	-2001.00	0.05
CattleT1	0.00	0.01	-0.38	0.70
CattleT5	0.02	0.03	0.55	0.58

GDP OLS model of 2013 (period of 2009—2013)

Coefficients

Variables	Estimate	Std. Error	t value	Pr(>|t|)
(Intercept)	6526.00	1487.00	4389.00	0.00
FCT1	0.00	0.00	-1342.00	0.18
Def	-0.04	0.15	-0.25	0.80
PastT5	-0.03	0.08	-0.38	0.71
SoyPT5	0.05	0.03	1988.00	0.05
SecMT5	0.00	0.00	-1059.00	0.29
LaborAgT1	1.72	1.49	1148.00	0.25
LaborAgT5	-0.90	4.67	-0.19	0.85
CattleT1	0.00	0.01	-0.76	0.45
CattleT5	0.06	0.03	1611.00	0.11

GDP OLS model of 2014 (period of 2010—2014)

Coefficients

Variables	Estimate	Std. Error	t value	Pr(>|t|)
(Intercept)	6638.00	2352.00	2823.00	0.01
FCT1	-0.01	0.00	-1478.00	0.14
Def	-0.01	0.20	-0.07	0.95
PastT5	0.00	0.10	0.03	0.97
SoyPT5	0.07	0.04	2022.00	0.05
SecMT5	0.01	0.02	0.68	0.50
LaborAgT1	3.07	2.44	1259.00	0.21
LaborAgT5	-4.50	7.06	-0.64	0.52
CattleT1	-0.02	0.01	-1772.00	0.08
CattleT5	0.04	0.05	0.77	0.44

GDP OLS model of 2015 (period of 2011—2015)

Coefficients

Variables	Estimate	Std. Error t	value	Pr(>|t|)
(Intercept)	6689.00	1379.00	4851.00	0.00
FCT1	0.00	0.00	-0.87	0.39
Def	0.01	0.11	0.10	0.92
PastT5	-0.01	0.06	-0.15	0.88
SoyPT5	0.02	0.02	1216.00	0.23
SecMT5	0.01	0.01	1206.00	0.23
LaborAgT1	0.31	1.38	0.22	0.82
LaborAgT5	0.37	4.67	0.08	0.94
CattleT1	0.00	0.01	-0.34	0.73
CattleT5	-0.01	0.03	-0.25	0.80

GDP OLS model of 2016 (period of 2012—2016)

Coefficients

Variables	Estimate	Std. Error	t value	Pr(>|t|)
(Intercept)	7632.00	1200.00	6361.00	0.00
FCT1	0.00	0.00	-0.86	0.39
Def	-0.01	0.09	-0.12	0.91
PastT5	0.04	0.05	0.77	0.44
soyPT1	0.01	0.00	2996.00	0.00
SoyPT5	0.05	0.01	3711.00	0.00
SecMT5	0.01	0.01	0.96	0.34
LaborAgT1	0.70	1.38	0.51	0.61
LaborAgT5	14.20	5.93	2394.00	0.02
CattleT1	0.00	0.00	-0.43	0.67
CattleT5	-0.03	0.02	-1116.00	0.27

GDP OLS model of 2017 (period of 2013—2017)

Coefficients

Variables	Estimate	Std. Error	t value	Pr(>|t|)
(Intercept)	6498.00	1250.00	5200.00	0.00
FCT1	0.00	0.00	0.32	0.75
Def	0.03	0.10	0.33	0.74
PastT5	-0.02	0.06	-0.27	0.79
SoyPT5	0.03	0.02	2321.00	0.02
SecMT5	0.01	0.01	0.95	0.34
LaborAgT1	0.55	1.21	0.46	0.65
LaborAgT5	11.06	5.10	2170.00	0.03
CattleT1	-0.01	0.00	-1836.00	0.07
CattleT5	-0.01	0.03	-0.40	0.69

GDP OLS model of 2017 (period of 2013—2017)

Coefficients

Variables	Estimate	Std. Error t	value	Pr(>|t|)
(Intercept)	5957.00	1712.00	3481.00	0.00
FCT1	0.00	0.00	0.23	0.82
Def	0.01	0.16	0.06	0.95
PastT5	0.00	0.10	-0.03	0.98
SoyPT5	0.08	0.03	2905.00	0.00
SecMT5	0.00	0.01	-0.30	0.77
LaborAgT1	0.48	1.43	0.34	0.74
LaborAgT5	13.47	4.72	2852.00	0.01
CattleT1	-0.01	0.01	-1529.00	0.13
CattleT5	-0.01	0.04	-0.25	0.81

SUPPLEMENTARY INFORMATION 4

Results of the augmented Dickey-Fuller (ADF) test. The ADF test was applied to our dependent and explanatory variables and we accepted the null-hypothesis, hence indicating the existence of unit root in their respective times series. Therefore, the variables of 'deforestation', 'pasture', 'soybean production', 'agricultural labor', 'maize second-crop production', 'cattle herd', and 'GDP per capita' were non-stationarity along the period between 2000 and 2018. The variable 'forest cover' was the only that the ADF test rejected null-hypothesis at p-value<0.01, indicating stationarity over the entire period.

Results of the Engle-Granger cointegration test. The test revealed absence of cointegration (i.e., accepting the null-hypothesis) between the two dependent variables when tested one by one with each of our explanatory variables, using the temporal series from 2000 to 2018.

Results of the Global Moran's I test. After applying the test for each of our model subsets (for Deforestation and GDP per capita models), to the case of the Deforestation model, no spatial autocorrelation was found just for the subsets of 2017 (2013—2017) and 2018 (2014—2018). However, to the GDP per capita model, the spatial autocorrelation was found just to the models of 2005 (2001—2005), 2006 (2002—2006), and 2007 (2003—2007).

Results of the Anselin Local Moran's I test. The analysis of the 'soybean production', 'deforestation', and 'GDP per capita' variables revealed a pattern of higher-higher cluster of municipalities in the central parts of the State for the 'soybean production', but unclear clustering patterns for the other two variables (Supplementary Fig. 1). This result reinforces the path dependence of soybean expansion within Mato Grosso state.

Results of the Likelihood Ratio test (LR). By applying the LR test we found that for all Deforestation model subsets the restrictions were not true, hence indicating the more complex model (i.e., Spatial Durbin Error Model—SDEM) having a better fit compared to an OLS (p-values<0.01 of significance). The only exceptions were to the subsets of 2017 and 2018, although with better (i.e., closer to zero) log likelihood results than the OLS. The log likelihood values for all GDP per capita model subsets were better (i.e., closer to zero) compared to OLS, but with significant LR at p-value<0.01 to only seven subsets out of fifteen (i.e., 2004, 2005, 2006, 2007, 2008, 2009, and 2013).

Results of the variation inflation factor (VIF). None of our Deforestation and GDP per capita model subsets scored over 5, therefore not exceeding the limit defined as acceptable by our methods. For the models were the full set of variables exceeded the VIF value of 5, the respective variable(s) over 5 were excluded (see results in Supplementary information 1, 2 and 3).

SUPPLEMENTARY FIGURES

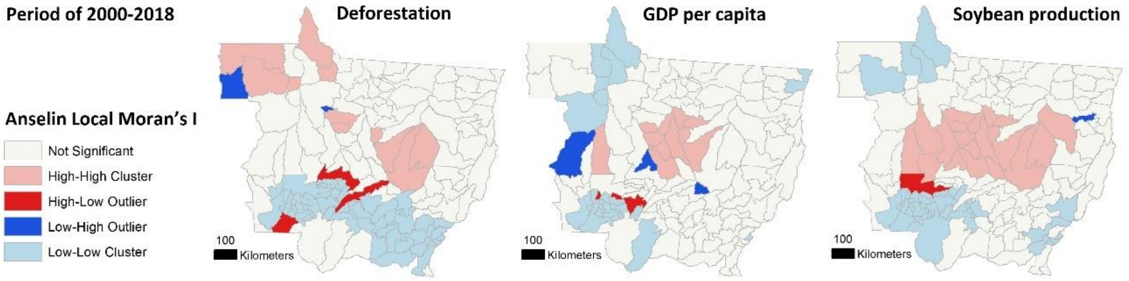

Supplementary Figure 1. The maps highlight the cluster and outlier spatial patterns according to the Anselin Local Moran's I test. The data for each test (Deforestation, GDP per capita, and Soybean production) are represented by the total changes between 2000 and 2018 at the municipality level.

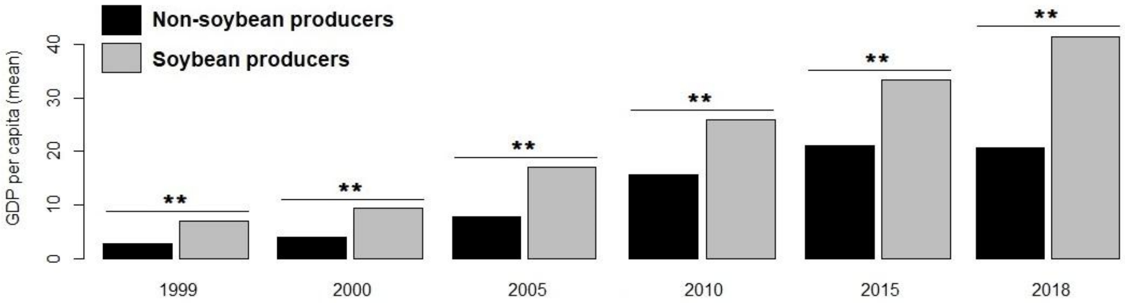

Supplementary Figure 2. Average values of GDP per capita according to municipalities grouped as soybean and non-soybean producers for the years of 1999, 2000, 2005, 2010, 2015, and 2018. Asterisks represent statistical significance of *Mann-Whitney U test* at *p-value* <0.05 * and *p-value* <0.01 **.

SUPPLEMENTARY TABLE

The freely available datasets used in this study are presented in **Supplementary Table 1**.

Supplementary Table 1. Datasets used to prepare dependent and independent variables.

Data	Source/table	Website
Statistics of cattle herd	IBGE/3939	https://sidra.ibge.gov.br/pesquisa/ppm/tabelas/
Soybean production	IBGE/1612	https://sidra.ibge.gov.br/pesquisa/pam/tabelas
GDP	IBGE/5938, 21	https://sidra.ibge.gov.br/pesquisa/pib-munic/tabelas
Population	IBGE/6579	https://sidra.ibge.gov.br/pesquisa/estimapop/tabelas
Maize second-crop production	IBGE/839	https://sidra.ibge.gov.br/pesquisa/pam/tabelas
Agricultural labor	MTE	https://bi.mte.gov.br/bgcaged/rais.php
Land-use/Land-cover	MapBiomas	https://mapbiomas.org/
Slope	UFRGS	http://www.ecologia.ufrgs.br/labgeo
